# New polychlorinated bibenzyls from *Rhododendron* *minutiflorum*

**DOI:** 10.1007/s13659-022-00364-x

**Published:** 2023-01-09

**Authors:** Yang-Li Zhu, Li Deng, Yu Tang, Xian-Zhe Fan, Yang Han, Mei Pan, Li-Jun Zhang, Hai-Bing Liao

**Affiliations:** 1 State Key Laboratory for Chemistry and Molecular Engineering of Medicinal Resources, Collaborative Innovation Center for Guangxi Ethnic Medicine, School of Chemistry and Pharmaceutical Sciences, Guangxi Normal University, Guilin, 541004 People’s Republic of China; 2 Guangxi Key Laboratory of Citrus Biology, Guangxi Academy of Specialty Crops, Guilin, 541004 People’s Republic of China; 3 Guilin Pharma Company, Guilin, 541007 People’s Republic of China

**Keywords:** *Rhododendronminutiflorum*, Polychlorinated bibenzyls, Matrine, Insecticidal activity, *Diaphorinacitri*, *α*-Glucosidase inhibitory activity, Acarbose

## Abstract

Five new polychlorinated bibenzyls (**1**–**5**) along with 3 known compounds (**6**–**8**) were isolated from the stems and leaves of *Rhododendron* *minutiflorum*. The chemical structures of all the isolates were determined by spectroscopic methods, and compounds **1** and **2** were further verified by single-crystal X-ray diffraction analyses. Compounds **1**–**5** were halogenated compounds which bear three to five chlorine atoms in their chemical structures. Biologically, compounds **2**, **5** and **6** showed varying degrees of toxicity toward the Asian citrus psyllid (*Diaphorina* *citri*) with LD_50_ values 27.15, 17.02 and 16.20 mg/L, respectively. These values were comparable to the positive control matrine (LD_50_ = 11.86 mg/L), which were calculated using observations on day 6. Meanwhile, compound **4** had *α*-glucosidase inhibitory activity with IC_50_ value of 17.87 ± 0.74 *μ*M.

**Graphical Abstract:**

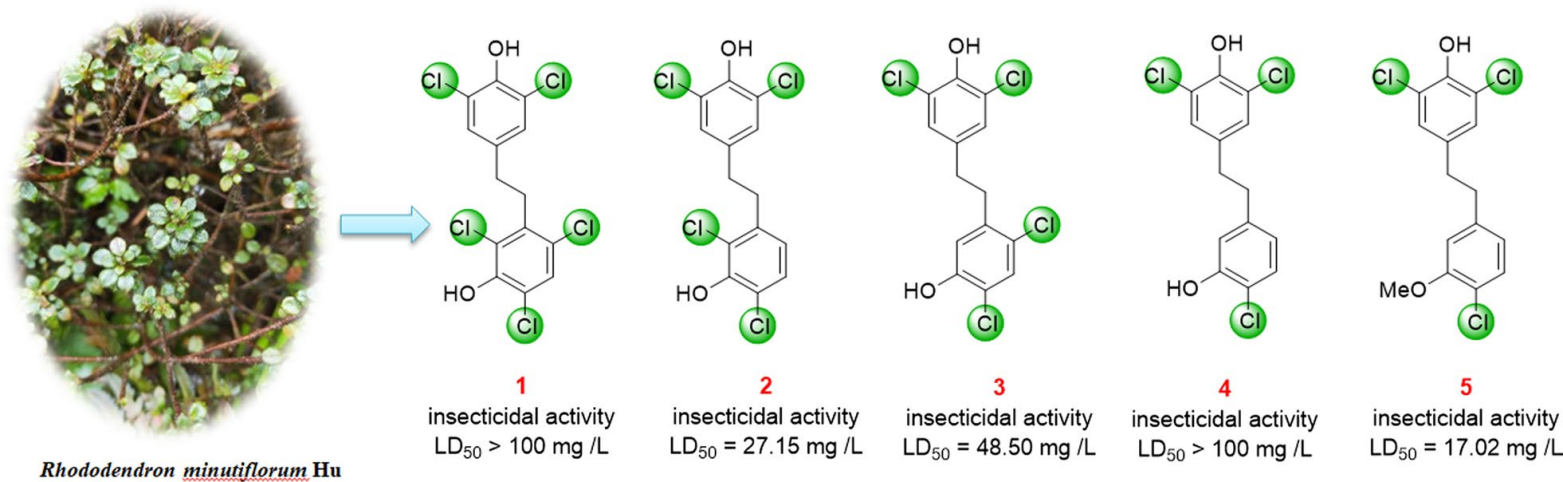

**Supplementary Information:**

The online version contains supplementary material available at 10.1007/s13659-022-00364-x.

## Introduction

Bibenzyl compounds are categorized as a class of secondary metabolite with parent nucleus structure that consists of two benzyl units which are linked by a C–C single bond at the benzylic position. Substituents on the benzene ring usually occur at the para- and meta-positions of the benzene ring and common substituents include methyl and hydroxyl groups [1]. Bibenzyl compounds and their derivatives which possessed a variety of biological activities including insecticidal [2], antifungal [3, 4], anti-inflammatory [5], anticancer [6, 7], neuroprotective [8] and other biological activities [9, 10] were discovered from a wide range of plants. Although a large number of halogenated compounds have been found in nature [11], natural halogenated bibenzyls are rarely isolated from higher plants.

**Fig. 1:**
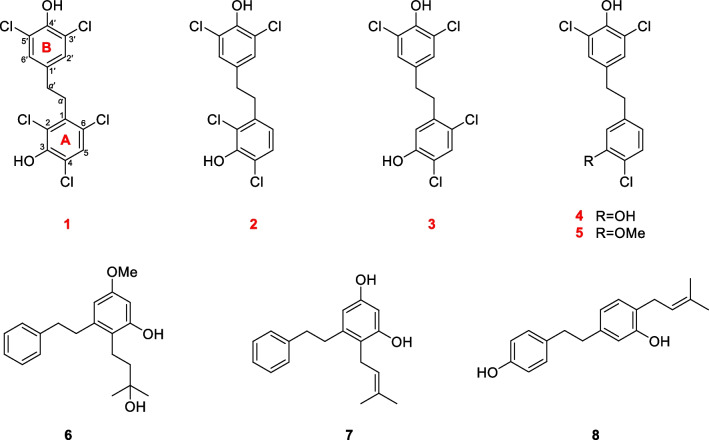
Chemical structures of compounds **1**–**8**

*Rhododendron*, which belongs to the family of Ericaceae, is one of the largest genera of vascular plants that is widely distributed all over the world, including the 542 species which grow in the mainland of China [12, 13]. A variety of compounds, such as terpenoids, lignans, and flavonoids [14–16] that have been identified from plants of this genus were reported with different biological activities [12, 13]. *Rhododendron* *minutiflorum* Hu, belonged to evergreen erect shrub, mainly distributed in the southern area of China. Previously, 2 omphalane-type sesquiterpenoids, 30 triterpenoids and their derivatives with potent *α*-glucosidase inhibitory activity have been isolated from *R.* *minutiflorum* by our group [13]. Moreover, partial species of *Rhododendron* exhibited insecticidal properties [12, 17]. In order to further study the phytochemical components and to analyze the biological activity, this work isolated five new polychlorobenzyls (**1**–**5**) and three known bibenzyls (**6**–**8**) (Fig. 1) from the leaves and stems of *R.* *minutiflorum*. The chemical structures of these compounds were determined by spectroscopic methods, whereas compounds **1** and **2** were further verified by single-crystal X-ray diffraction analyses. Subsequently, the *α*-glucosidase inhibitory activities and the insecticidal properties of these compounds were evaluated.

## Results and discussion

The air-dried stems and leaves of *R.* *minutiflorum* were extracted with 95% EtOH-H_2_O and then the organic solvent was removed under vacuum to obtain EtOH extract. Subsequently, the EtOH extract was suspended with an appropriate amount of distilled water and extracted with EtOAc for 3 times. The organic layer was concentrated to obtain the EtOAc part (900 g). The EtOAc part was further applied to various chromatographic columns (CC) such as Diaion HP20 macroporous resin, silica gel, Sephadex LH-20, reversed-phase C18 and semi-preparative RP-HPLC to obtain 8 different compounds. Among these, compounds **1**–**5** were undescribed halogenated compounds with multiple chlorine atoms. Based on spectroscopic analysis and comparison with previous literature data, 3 known compounds were determined as: 3-hydroxyl-5-methoxy-2-(3′-hydroxyl-3′-methylbutyl)-bibenzyl (**6**) [18], 3,5-dihydroxyl-2-(3′-methyl-2′-butenyl)-bibenzyl (**7**) [18], 3,4″-dihydroxyl-4-(3′-methyl-2′-butenyl)-bibenzyl (**8**) [19], respectively. All these compounds were isolated from *R.* *minutiflorum* for the first time.

### Structural identification of compounds

**Fig. 2:**
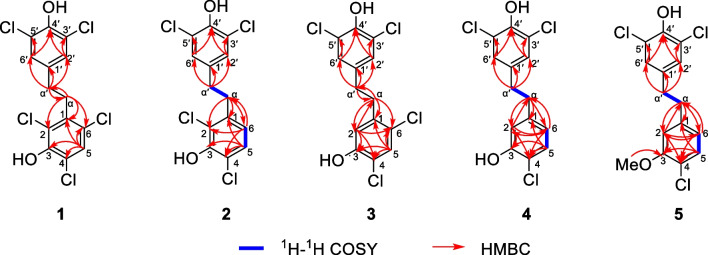
The key ^1^H–^1^H COSY and HMBC correlations for compounds **1**–**5**

**Fig. 3:**
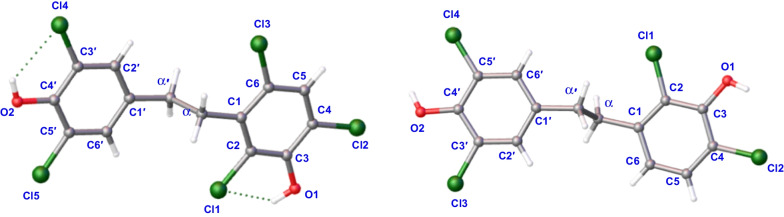
X-ray ORTEP drawing of **1** and **2**

Compound **1**, colorless crystals, has a molecular formula of C_14_H_9_Cl_5_O_2_ with eight degrees of unsaturation based on its ^1^H and ^13^C NMR data and the (–) HRESIMS [M−H]^−^ ion at *m/z* 382.8992 (calcd 382.8972). IR absorption bands confirmed the hydroxy (3417 cm^−1^) and aromatic ring (1453, 1395 cm^−1^) functionalities in compound **1**. The NMR data (Tables 1 and 2) showed characteristic signals for two phenyl rings [*δ*_H_ 7.35 (1H, s), 7.14 (2H, s); *δ*_C_ 119.3–147.2] and two *sp*^3^ methylene groups [*δ*_H_ 3.11 (2H, m), 2.71 (2H, m); *δ*_C_ 33.8, 33.0]. According to the key HMBC correlations from H-2′/H-6′ to C-4′ and C-3′/C-5′, H-*α*′ to C-*α*, C-1′ and C-2′/C-6′ (Fig. 2), a 3′, 4′, 5′ trisubstituted benzyl skeleton moiety was expounded. Moreover, the 2,3,4,6-tetrasubstituted benzyl skeleton moiety of **1** was further deduced by the HMBC correlations from H-5 to C-1, C-3, C-4 and C-6, H-*α* to C-*α*′, C-1, C-2 and C-6, indicating that **1** belonged to the bibenzyl compounds. Based on the molecular formula, the positions of the five chlorine atoms and the two hydroxyl groups could not determine. By analyzing the NMR data of bibenzyl compounds previously reported in the literature, the chemical shift of aromatic carbon with hydroxyl group was above 145 ppm [18]. Therefore, it was inferred to that aromatic quaternary carbons with chemical shifts of *δ*_C_ 147.2 and *δ*_C_ 146.4 were linked to hydroxyl groups, while the other aromatic quaternary carbons were attached to the chlorine atoms. Finally, the single-crystal X-ray crystallographic study (Fig. 3) confirmed the positions for hydroxyl groups at C-3 and C-4′ and chlorine atoms at C-2, C-4, C-6, C-3′ and C-5′. Consequently, compound **1** was assigned as 2,4,6,3′,5′-pentachloro-3,4′-dihydroxybibenzyl.

Compound **2** was obtained as colorless crystals and its molecular formula was C_14_H_10_Cl_4_O_2_ with eight degrees of unsaturation according to its (−) HRESIMS [M−H]^−^ ion at *m/z* 348.9391 (calcd 348.9362) and the NMR data. From the IR absorption bands, compound **2** contains hydroxy (3316 cm^−1^) and aromatic ring (1419 cm^−1^) functionalities. The NMR data (Tables 1 and 2) indicated the characteristic signals for two methylene groups [*δ*_H_ 2.94 (2H, m), 2.78 (2H, m); *δ*_C_ 35.7, 34.6] and two benzene rings [*δ*_H_ 7.17 (1H, d, *J* = 8.4 Hz), 7.07 (2H, s), 6.66 (1H, d, *J* = 8.4 Hz); *δ*_C_ 119.0–148.1]. The ^1^H-^1^H COSY correlations (Fig. 2) of **2** showed two spin systems of H-5/H-6 and H-*α*/H-*α*′. The key HMBC correlations (Fig. 2) from H-*α* to C-1, C-2 and C-6, H-5 to C-3, C-4 and C-1, H-6 to C-*α*, C-2 and C-4 and the chemical shifts further verified the presence of a 2,3,4-trisubstituted benzyl skeleton and the positions for hydroxyl group at C-3 and chlorine atoms at C-2 and C-4. The other HMBC correlations from *δ*_H_ 2.78 (H-*α*′) to *δ*_C_ 128.3 (C-2′, 6′) and *δ*_C_ 134.5 (C-1′), *δ*_H_ 7.07 (H-2′, 6′) to *δ*_C_ 146.3 (C-4′) and *δ*_C_ 120.9 (C-3′, 5′) supported that the positions of C-3′, C-5′ and C-4′ were substituted by two chlorine atoms and hydroxyl group, respectively. Furthermore, detailed analysis of the NMR data and molecular formula revealed that the absence of a chlorine atom at C-6 in **2** than **1**. Finally, the structure of **2** was further verified by single-crystal X-ray diffraction analysis with Flack parameter of − 0.008 (14) (Fig. 3), and the structure of compound **2** was deduced as 2,4,3′,5′-tetrachloro-3,4′-dihydroxybibenzyl, as shown in Fig. 1.

Compound **3**, a white amorphous powder, had a molecular formula of C_14_H_10_Cl_4_O_2_, with eight degrees of unsaturation, deduced from its NMR data and the (−) HRESIMS [M−H]^−^ ion at *m/z* 348.9390 (calcd 348.9362). The detailed NMR data (Tables 1 and 2) analysis revealed that the structure of **3** was similar to **2**, with only a change in position of the chlorine atom at C-2 in **2** to C-6 in **3**. In the HMBC spectrum (Fig. 2), the correlations from *δ*_H_ 2.89 (H-*α*) to *δ*_C_ 34.7 (C-*α*′), 117.7 (C-2), 139.1 (C-1), 134.5 (C-1′) and 125.3 (C-6), *δ*_H_ 6.82 (H-2) to *δ*_C_ 35.4 (C-*α*), 150.3 (C-3), 118.4 (C-4) and 125.3 (C-6), *δ*_H_ 7.34 (H-5) to *δ*_C_ 139.1 (C-1), 150.3 (C-3), 118.4 (C-4) and 125.3 (C-6) can confirm the above conclusion. From the perspective of biosynthesis, the locations for hydroxyl group at C-3 and chlorine atom at C-4 could be presumed. Therefore, the structure of **3** was defined as shown in Fig. 1 and named as 4,6,3′,5′-tetrachloro-3,4′-dihydroxybibenzyl.

Compound **4** was obtained as a white amorphous powder and had a molecular formula of C_14_H_11_Cl_3_O_2_ based on the (−) HRESIMS [M−H]^−^ ion at *m/z* 314.9781 (calcd 314.9752) and NMR data. The NMR data (Tables 1 and 2) indicated characteristic signals for two phenyl rings [*δ*_H_ 7.21 (1H, d, *J* = 8.4 Hz), 7.04 (2H, s), 6.82 (1H, d, *J* = 2.0 Hz), 6.64 (1H, dd, *J* = 8.4, 2.0 Hz); *δ*_C_ 151.3–116.2] and two methylene groups [*δ*_H_ 2.79 (4H, m); *δ*_C_ 37.2, 36.4]. The NMR data suggested that **4** was a bibenzyl compound. The ^1^H–^1^H COSY correlations (Fig. 2) of **4** showed two spin systems of H-5/H-6 and H_2_-*α*/H_2_-*α*′. Moreover, the key HMBC correlations (Fig. 2) supported that the positions of C-3, C-4, C-3′, C-4′ and C-5′ in **4** were substituted by two hydroxyl groups and three chlorine atoms. The aforementioned analyses suggested that the structure of **4** closely resembled **2**, except for the absence of a chlorine atom at C-2 in **4**. Thus, **4** was characterized as a polychlorinated bibenzyl and assigned as 4,3′,5′-trichloro-3,4′-dihydroxybibenzyl.

Compound **5**, a white amorphous powder, had a molecular formula of C_15_H_13_Cl_3_O_2_ with eight degrees of unsaturation deduced from its NMR data and the (−) HRESIMS [M−H]^−^ ion at *m/z* 328.9931 (calcd 328.9908). The detailed analyses of 1D NMR data (Tables 1 and 2) and 2D NMR data (Fig. 2) showed that the structure of **5** was similar to **4**, differing only by the hydroxy at C-3 in **4** being replaced by a methoxy in **5**, which was confirmed by the HMBC correlation (Fig. 2) from *δ*_H_ 3.86 (–OMe) to *δ*_C_ 155.0 (C-3). Thus, the structure of **5** was elucidated as shown in Fig. 1 and named as 4,3′,5′-trichloro-4′-hydroxyl-3-methoxybibenzyl.

### Insecticidal activity and *α*-glucosidase inhibitory activity

**Fig. 4:**
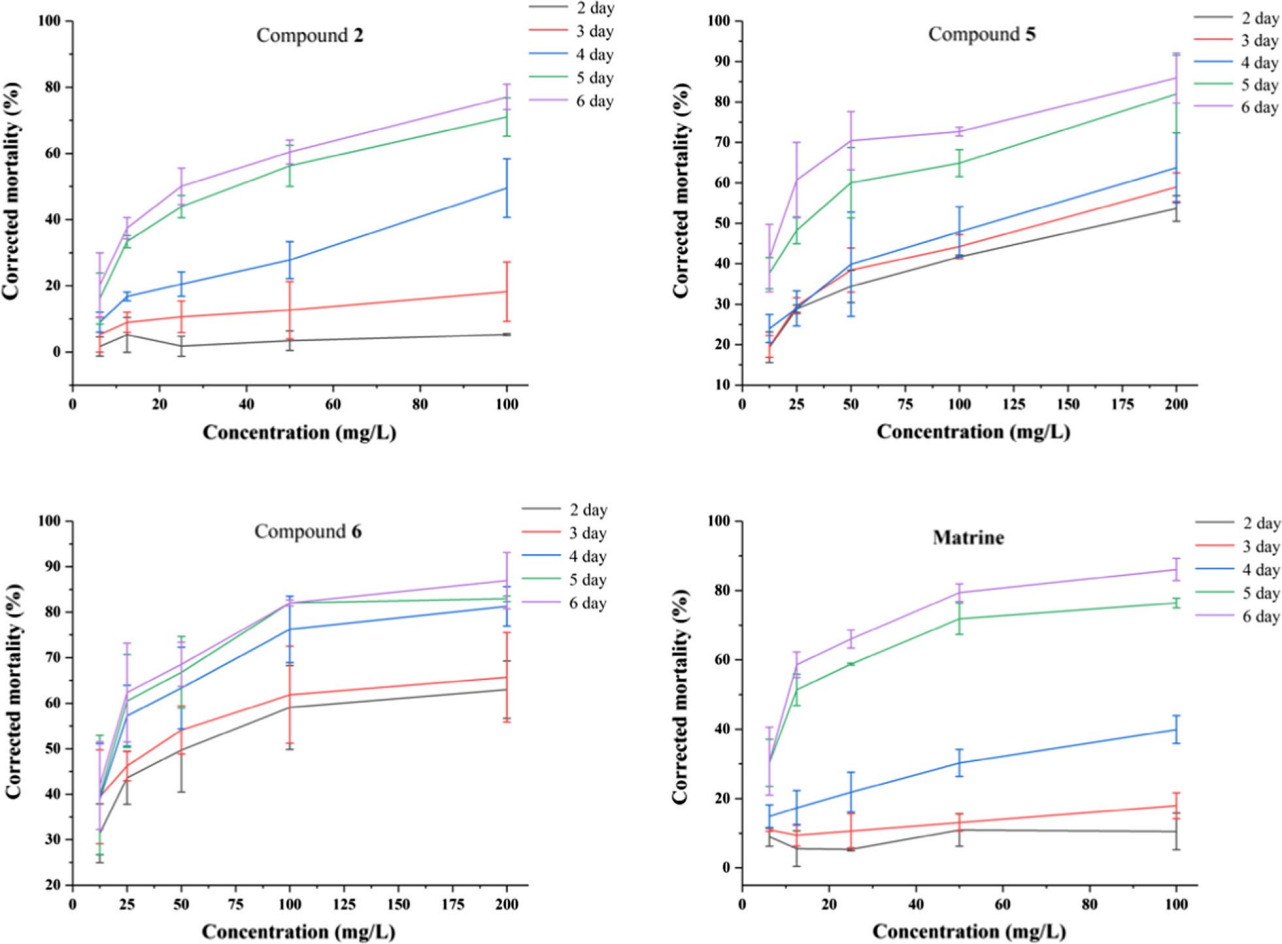
Toxic activity anainst *D.* *citri* of compounds **2**, **5**, **6** and matrine

In order to evaluate the insecticidal activities of all the isolated compounds, their toxicities on *D.* *citri* Kuwayama were tested. With compound **4** as the only exception, compounds **1**–**3** and **5**–**8** exhibited different toxicities to *D.* *citri* compared to the 10% methanol or DMSO controls. Compounds **2**, **5** and **6** showed remarkable insecticidal activities, with LD_50_ values of 27.15, 17.02 and 16.20 mg/L, respectively. While the LD_50_ of matrine was 11.86 mg/L (Table 3). Interestingly, on the 4th day after the test started, the corrected mortality rate of citrus psyllid is higher than 50% when the compound concentration is higher than 50 mg/L, indicating that compounds **2**, **5** and **6** had insecticidal activity comparable to positive control matrine (Fig. 4). These data could provide reference and scientific basis for the further development and utilization of new pesticides against the Asian citrus psyllid *D.* *citri*. Besides, the *α*-glucosidase inhibitory activity of compounds **1**–**8** were detected by PNPG method, with acarbose served as a positive control (Table 4). The results showed that compounds **1**, **3**–**8** gave different degrees of inhibitory activity with IC_50_ values from 17.87 to 82.84 *μ*M (the IC_50_ of acarbose was 3.07 × 10^–3^* μ*M).

**Table 1:** ^13^C NMR data of compounds **1**–**5**

No.	**1**^a^	**2**^a^	**3**^a^	**4**^a^	**5**^a^
	*δ*_C_, type	*δ*_C_, type	*δ*_C_, type	*δ*_C_, type	*δ*_C_, type
1	136.4, s	138.5, s	139.1, s	141.8, s	141.0, s
2	122.1, s	121.1, s	117.7, d	116.2, d	112.6, d
3	147.2, s	148.1, s	150.3, s	151.3, s	155.0, s
4	119.3, s	119.0, s	118.4, s	117.7, s	120.3, s
5	128.4, d	127.6, d	129.3, d	128.9, d	130.2, d
6	125.8, s	122.0, d	125.3, s	121.7, d	121.4, d
α	33.8, t	35.7, t	35.4, t	37.2, t	37.6, t
α′	33.0, t	34.6, t	34.7, t	36.4, t	36.6, t
1′	134.4, s	134.5, s	134.5, s	134.8, s	134.8, s
2′	128.3, d	128.3, d	128.3, d	128.3, d	128.3, d
3′	121.0, s	120.9, s	121.0, s	120.9, s	120.9, s
4′	146.4, s	146.3, s	146.3, s	146.1, s	146.2, s
5′	121.0, s	120.9, s	121.0, s	120.9, s	120.9, s
6′	128.3, d	128.3, d	128.3, d	128.3, d	128.3, d
3-OMe					56.2, q

^a^In CDCl_3_, NMR at 100 MHz

**Table 2:** ^1^H NMR data of compounds **1**–**5**

No.	**1**^a^	**2**^a^	**3**^a^	**4**^a^	**5**^a^
	*δ*_H_ mult. (*J*)	*δ*_H_ mult. (*J*)	*δ*_H_ mult. (*J*)	*δ*_H_ mult. (*J*)	*δ*_H_ mult. (*J*)
2			6.82, s	6.82, d (2.0)	6.66, d (2.0)
5	7.35, s	7.17, d (8.4)	7.34, s	7.21, d (8.4)	7.25, d (7.6)
6		6.66, d (8.4)		6.64, dd (8.4, 2.0)	6.68, dd (7.6, 2.0)
α	3.11, m	2.94, m	2.89, m	2.79, m^b^	2.82, m^b^
α′	2.71, m	2.78, m	2.77, m	2.79, m^b^	2.82, m^b^
2′	7.14, s	7.07, s	7.08, s	7.04, s	7.05, s
6′	7.14, s	7.07, s	7.08, s	7.04, s	7.05, s
3-OMe					3.86, s

^a^In CDCl_3_, NMR at 400 MHz; ^b^Overlapped signals are reported without designating multiplicity

**Table 3:** Toxicity of compounds **2**, **3**, **5**–**7** and matrine against *D.* *citri* during 6 days after treatment

Compound	Regression equation	LD_50_ (mg/L)	95% confidence interval	R
**2**	y = 3.2850 + 1.1961x	27.15	23.8790–30.8889	0.9934
**3**	y = 2.9190 + 1.2345x	48.50	33.0602–93.0829	0.9855
**5**	y = 3.8239 + 0.9554x	17.02	6.4811–27.5943	0.9722
**6**	y = 3.6959 + 1.0780x	16.20	6.9766–25.3504	0.9871
**7**	y = 3.2422 + 1.0419x	48.64	31.5027–106.5387	0.9850
Matrine	y = 3.6584 + 1.2493x	11.86	8.7628–16.0413	0.9773

**Table 4:** Inhibitory effects of compounds **1** and **3**–**8** on *α*-glucosidase (mean ± SE)

Compound	IC_50_ (*μ*M)	Compound	IC_50_ (*μ*M)
**1**	82.84 ± 4.99	**6**	76.05 ± 11.99
**3**	60.44 ± 1.54	**7**	56.74 ± 1.42
**4**	17.87 ± 0.74	**8**	65.44 ± 2.04
**5**	62.70 ± 6.87	Acarbose	3.07 × 10^–3^

Furthermore, the preliminary structure–activity relationships (SAR) analyses showed that compounds with two chlorine atoms at C-2 and C-4 or C-4 and C-6 in ring A possessed insecticidal activity while compounds with three chlorine atoms at C-2, C-4 and C-6 in ring A had no more insecticidal activity, such as the insecticidal activity of **2**, **3** > **1**. Besides, if there was only one chlorine atom in ring A, the insecticidal activity was decreased (**2**, **3** > **4**); however, methoxylation of C-3 can significantly increase the insecticidal activity (**5** > **4**).

## Experimental

### General experimental procedures

The instruments, materials and reagents used in this study were consistent with those reported in the literature [13, 20].

### Plant materials

Dr. Shao-Qing Tang of Guangxi Normal University, Guangxi Province, People’s Republic of China identified the stems and leaves of *Rhododendron* *minutiflorum* that were collected from Wuming County, Guangxi Province, in May 2019. The voucher specimen (No. TS-20190505) was deposited at the State Key Laboratory of Chemistry and Molecular Engineering of Medicinal Resources, Guangxi Normal University.

### Extraction and isolation

The air-dried stems and leaves of *R.* *minutiflorum* (13.4 kg) were extracted with 95% EtOH-H_2_O (4 × 70 L, each 3 h) heating reflux to obtain the EtOH extract. The extract was concentrated under reduced pressure and then suspended with an appropriate amount of distilled water. Subsequently, the suspension liquid was extracted by EtOAc for 3 times and concentrated to generate the EtOAc part (900 g). The EtOAc part was subjected to a Diaion HP20 macroporous resin column eluting with EtOH-H_2_O (from 30:70 to 95:5) and acetone to yield five fractions (A–E).

Fraction D (12.0 g) was separated by silica gel column chromatography and eluted with CH_2_Cl_2_–MeOH (from 100:1 to 1:1) with increasing polarity to yield ten fractions (D1–D10). Fraction D1 (1.9 g) was chromatographed on a reversed-phase C18 (RP-C18) column eluting with MeOH–H_2_O (from 60:40 to 100:0), yielding fourteen subfractions (D1f1–D1f14). The subfraction D1f3 (159.0 mg) was separated by silica gel (200–300 mesh) column chromatography (CC) and eluted with PE/EtOAc (from 10:1 to 1:1) with increasing polarity and then purified by semi-preparative RP-HPLC (MeOH–H_2_O, 65:35) to generate compound **2** (72.2 mg, *t*_R_ = 156.8 min). The subfraction D1f2 (118.0 mg) was separated by silica gel CC, and followed by purification by semi-preparative RP-HPLC (MeOH–H_2_O, 65:35) to generate **4** (5.6 mg, *t*_R_ = 67.3 min). The subfraction D1f4 (142.0 mg) was separated by silica gel CC and eluted with PE/EtOAc (from 10:1 to 1:1) and purified by semi-preparative RP-HPLC (MeOH–H_2_O, 68:32) to generate **3** (5.1 mg, *t*_R_ = 128.0 min).

Fraction E (75.3 g) was separated by silica gel CC and eluted with CH_2_Cl_2_–MeOH (from 100:1 to 1:1) with increasing polarity to yield nine fractions (E1–E9). Fraction E1 (16.1 g) was chromatographed on a RP-C18 column eluting with MeOH–H_2_O (from 55:45 to 100:0), yielding twenty-one subfractions (E1k1–E1k21). The subfractions E1k7 (98.0 mg) and E1k6 (259.8 mg) were respectively separated by Sephadex LH-20 column (MeOH) and purified by semi-preparative RP-HPLC (MeCN–H_2_O, 55:45, 50:50, respectively) to generate **1** (7.6 mg, *t*_R_ = 81.8 min) and **5** (5.1 mg, *t*_R_ = 180.0 min).

Fraction C (100 g) was separated by silica gel CC and eluted with CH_2_Cl_2_–MeOH (from 100:1 to 1:1) with increasing polarity to yield fifteen fractions (C1–C15). The fraction C5 (2.2 g) was chromatographed on a RP-C18 column eluting with MeOH–H_2_O (from 50:50 to 100:0), yielding thirteen subfractions (C5h1–C5h13). The subfraction C5h5 (61.0 mg) was purified by semi-preparative RP-HPLC (MeCN–H_2_O, 45:55) to generate **6** (15.7 mg, *t*_R_ = 67.8 min) and **7** (28.2 mg, *t*_R_ = 127.7 min). The subfraction C5h6 (152.0 mg) was separated by Sephadex LH-20 and purified by semi-preparative RP-HPLC (MeCN–H_2_O, 45:55) to generate **8** (5.0 mg, *t*_R_ = 182.8 min).

#### 2,4,6,3′,5′-Pentachloro-3,4′-dihydroxybibenzyl (1)

Colorless crystals; m.p. 151–152 ℃; UV (MeOH) *λ*_max_ (log *ε*) 222 (3.99), 290 (3.28) nm; IR (KBr) *ν*_max_ 3672, 3417, 2975, 1453, 1395, 1232, 1503, 879 cm^−1^; ^1^H (400 MHz) and ^13^C (100 MHz) NMR (in CDCl_3_) data, see Tables 1 and 2; (−) HRESIMS *m/z* 382.8992 [M−H]^−^, (calcd C_14_H_8_Cl_5_O_2_: 382.8972).

#### 2,4,3′,5′-Tetrachloro-3,4′-dihydroxybibenzyl (2)

Colorless crystals; m.p. 114–115 ℃; UV (MeOH) *λ*_max_ (log *ε*) 205 (5.19), 225 (4.78), 285 (4.17) nm; IR (KBr) *ν*_max_ 3316, 2976, 1419, 1049, 881, 799 cm^−1^; ^1^H (400 MHz) and ^13^C (100 MHz) NMR (in CDCl_3_) data, see Tables 1 and 2; (−) HRESIMS *m/z* 348.9391 [M−H]^−^, (calcd C_14_H_9_Cl_4_O_2_: 348.9362).

#### 4,6,3′,5′-Tetrachloro-3,4′-dihydroxybibenzyl (3)

White powder; UV (MeOH) *λ*_max_ (log *ε*) 227 (3.30), 284 (2.61) nm; IR (KBr) *ν*_max_ 3664, 3303, 2975, 1407, 1252, 1053, 983 cm^−1^; ^1^H (400 MHz) and ^13^C (100 MHz) NMR (in CDCl_3_) data, see Tables 1 and 2; (−) HRESIMS *m/z* 348.9390 [M−H]^−^, (calcd C_14_H_9_Cl_4_O_2_: 348.9362).

#### 4,3′,5′-Trichloro-3,4′-dihydroxybibenzyl (4)

White powder; UV (MeOH) *λ*_max_ (log *ε*) 226 (3.41), 275 (2.77) nm; IR (KBr) *ν*_max_ 3391, 2923, 1668, 1575, 1486, 1150, 1048, 874, 800 cm^−1^; ^1^H (400 MHz) and ^13^C (100 MHz) NMR (in CDCl_3_) data, see Tables 1 and 2; (−) HRESIMS *m/z* 314.9781 [M−H]^−^, (calcd C_14_H_10_Cl_3_O_2_: 314.9752).

#### 4,3′,5′-Trichloro-4′-hydroxyl-3-methoxybibenzyl (5)

White powder; UV (MeOH) *λ*_max_ (log *ε*) 226 (2.94), 279 (1.09) nm; IR (KBr) *ν*_max_ 3366, 2978, 1683, 1456, 1205, 1049, 881, 802 cm^−1^; ^1^H (400 MHz) and ^13^C (100 MHz) NMR (in CDCl_3_) data, see Tables 1 and 2; (−) HRESIMS *m/z* 328.9931 [M−H]^−^, (calcd C_15_H_12_Cl_3_O_2_: 328.9908).

#### 3,4″-Dihydroxyl-4-(3′-methyl-2′-butenyl)-bibenzyl (8)

Yellow oil; ^1^H NMR (600 MHz, MeOH-*d*_4_): *δ*_H_ 6.96 (2H, m, H-2′′/6′′), 6.89 (1H, d, *J* = 7.8 Hz, H-5), 6.66 (2H, m, H-3′′/5′′), 6.56 (1H, overlapped, H-2), 6.55 (1H, overlapped, H-6), 5.29 (1H, m, H-2′), 3.23 (2H, d, *J* = 7.2 Hz, H_2_-1′), 2.74 (4H, overlapped, H_2_-*α*, H_2_-*β*), 1.72 (3H, s, H_3_-4′), 1.70 (3H, s, H_3_-5′); ^13^C NMR (150 MHz, MeOH-*d*_4_): *δ*_C_ 156.4 (s, C-4′′), 155.8 (s, C-3), 142.0 (s, C-1), 134.2 (s, C-1′′), 132.6 (s, C-3′), 130.4 (d, C-2′′/6′′), 130.2 (d, C-5), 126.5 (s, C-4), 124.3 (d, C-2′), 120.7 (d, C-6), 116.0 (d, C-2/3′′/5′′), 39.2 (t, C-*α*), 38.4 (t, C-*β*), 28.9 (t, C-1′), 25.9 (q, C-4′), 17.8 (q, C-5′); ( +) HRESIMS *m/z* 283.1688 [M + H]^+^, (calcd C_19_H_23_O_2_: 283.1693) (Additional file 1: Fig. S58).

#### Single‑crystal X‑ray diffraction data of 1

Colorless crystals of **1**: Moiety formula were C_14_H_9_Cl_5_O_2_ and H_2_O(2), *M* = 422.49, monoclinic, space group *I*2/a, unit cell dimension *a* = 27.1575(11) Å, *b* = 4.3186(3) Å, *c* = 30.0170(12) Å, *α* = 90.0°, *β* = 91.182(4)°, *γ* = 90°, *V* = 3519.9(3) Å^3^, Z = 8, *T* = 293 K, *μ*(Cu Kα) = 7.655 mm^−1^, *Dc* = 1.594 g/cm^3^, *F*(000) = 1712.0. A total of 24,050 reflections were collected in the range 5.89° ≤ 2θ ≤ 134.15° with 3111 independent reflections [*R*_int_ = 0.1191, *R*_sigma_ = 0.0698]. The final *R* indexes [I ≥ 2σ (I)], *R*_1_ = 0.0710, *wR*_2_ = 0.1946, final *R* indexes [all data], *R*_1_ = 0.1132, *wR*_2_ = 0.2257. The goodness of fit on *F*^2^ was 1.125.

#### Single‑crystal X‑ray diffraction data of 2

Colorless crystals of **2**: Molecular formula was C_14_H_10_Cl_4_O_2_, *M* = 352.02, orthorhombic, space group *P*212121, unit cell dimension *a* = 4.7405(13) Å, *b* = 12.6052(5) Å, *c* = 24.1973(7) Å, *α* = *β* = *γ* = 90°, *V* = 1445.91(8) Å^3^, *Z* = 4, *T* = 293 K, *μ*(Cu Kα) = 7.423 mm^−1^, *Dc* = 1.617 g/cm^3^, *F*(000) = 721.0. A total of 6362 reflections were collected in the range 7.306° ≤ 2θ ≤ 134.126° with 2567 independent reflections [*R*_int_ = 0.0471, *R*_sigma_ = 0.0556]. The final *R* indexes [I ≥ 2*σ* (I)], *R*_1_ = 0.0330, *wR*_2_ = 0.0744, final *R* indexes [all data], *R*_1_ = 0.0386, *wR*_2_ = 0.0768. The goodness of fit on *F*^2^ was 0.991. Flack parameter: − 0.008(14).

Compounds **1** and **2** are colorless crystals that were obtained by vapor diffusion in MeOH solvent at room temperature. The intensity data were acquired in the way described in the previous literatures [21–23]. These data have been deposited in the Cambridge Crystallographic Data Centre as supplementary publications numbers CCDC 2105148 for **1**, 2105091 for **2**. Free copies of the data can be obtained from CCDC through www.ccdc.cam.ac.uk.

### The biological assay

In November 2021, *D.* *citri* were captured from 5-year-old citrus trees (*Citrus* *reticulata* *Blanco* *CV’* Shatangju) in an orchard in Lingchuan, Guilin, China (25° 41 N, 110° 36 E). To avoid accidental injury, all the *D.* *citri* were carefully collected and cultured. The insecticidal activities of eight compounds were evaluated using methods described in the literature [24, 25] with slightly modified. In brief, *D.* *citri* were treated with compounds **1**–**8** and the positive control at concentrations of 100, 50, 25, 12.5 and 6.25 mg/L or 200, 100, 50, 25 and 12.5 mg/L. The blank solution was prepared with 10% methanol or DMSO in distilled water, respectively. Finally, mortality was calculated every 24 h starting on the second day of the trial. The toxicity data were statistically analyzed by Probit analyses, and the value of 50% lethal dose (LD_50_) were calculated.

According to the previously reported method [13], all the isolated compounds were screened for *α*-glucosidase inhibitory activity. Acarbose (Med Chem Express, NJ, USA, HY-B0089) was used as a positive control.

## Conclusion

Five new polychlorinated bibenzyls (**1**–**5**) and three known bibenzyls (**6**–**8**) were isolated from the stems and leaves of *R.* *minutiflorum*. All the chemical structures were determined by spectroscopic methods, and the structures of compounds **1** and **2** were further verified by the single-crystal X-ray diffraction analyses. Compounds **1**–**5** were rare halogenated compounds which bear three to five chlorine atoms. As we know, it’s the first time to find polychlorinated bibenzyls from *Rhododendron* plants. The insecticidal activity against the Asian citrus psyllid *D.* *citri* and the inhibitory activity on *α*-glucosidase of all the isolated compounds were evaluated. It is concluded that compounds **2**, **5** and **6** from *R.* *minutiflorum* Hu exhibited potential insecticidal activity against the Asian citrus psyllid *D.* *citri*, and compounds **4** showed potent inhibitory activity against *α*-glucosidase.

## Supplementary Information

**Additional file 1: Figure S1. **Chemical structures ofcompounds **1**-**8** isolated from *R.minutiflorum* Hu.**Figure S2.** Comparison of HPLC-MS of compound **2 **and the EtOH extract from *R. minutiflorum*Hu*. ***Figure S3.** Comparison of HPLC-MS of compounds **1**-**5 **and the EtOH extract from *R. minutiflorum*Hu. **Table S1.** X-rayCrystallographic Data for Compound **1**. **Table S2.** X-ray CrystallographicData for Compound **2**. **Figure S4.** ^1^HNMR Spectrum of Compound **1** in CDCl_3_. **Figure S5.** ^13^C NMR Spectrum of Compound **1** in CDCl_3_. **Figure S6.** HSQCSpectrum of Compound **1** in CDCl_3_. **Figure S7.** HMBC Spectrum of Compound **1** in CDCl_3_. **Figure S8.** (-)Total HRESIMS Spectrum of Compound **1**. **Figure S9.** (-) Partial HRESIMS Spectrum of Compound **1**. **Figure S10.** UV Spectrum of Compound **1**. **Figure S11.** IR(KBr disc) Spectrum of Compound **1**. **Figure S12.** ^1^HNMR Spectrum of Compound **2** in CDCl_3_. **Figure S13.** ^13^C NMR Spectrum of Compound **2** in CDCl_3_. **Figure S14.** ^1^H-^1^H COSY Spectrum of Compound **2 **in CDCl_3_. **Figure S15.** HSQCS pectrum of Compound **2** in CDCl_3_. **Figure S16.** HMBC Spectrum of Compound **2** in CDCl_3_. **Figure S17.** (-)Total HRESIMS Spectrum of Compound **2**. **Figure S18.** (-) Partial HRESIMS Spectrum of Compound **2**. **Figure S19.** UVSpectrum of Compound **2**. **Figure S20.** IR (KBr disc) Spectrum of Compound **2**. **Figure S21.** ^1^H NMR Spectrum of Compound **3** in CDCl_3_. **Figure S22.** ^13^C NMR Spectrum of Compound **3** in CDCl_3_. **Figure S23.** HSQCSpectrum of Compound **3** in CDCl_3_. **Figure S24.** HMBC Spectrum of Compound **3 **in CDCl_3_. **Figure S25.** (-)Total HRESIMS Spectrum of Compound **3**. **Figure S26.** (-) Partial HRESIMS Spectrum of Compound **3**.Figure S27. UV Spectrum of Compound **3**. **Figure S28.** IR(KBr disc) Spectrum of Compound **3**. **Figure S29.** ^1^H NMR Spectrum of Compound **4** in CDCl_3_. **Figure S30.** ^13^C NMR Spectrum of Compound **4** in CDCl_3_. **Figure S31. **^1^H-^1^H COSY Spectrum of Compound **4 **in CDCl_3_. **Figure S32.** HSQCSpectrum of Compound **4** in CDCl_3_. **Figure S33.** HMBC Spectrum of Compound **4** in CDCl_3_. **Figure S34.** (-)Total HRESIMS Spectrum of Compound **4**. **Figure S35.** (-) Partial HRESIMS Spectrum of Compound **4**. **Figure S36.** UV Spectrum of Compound **4**. **Figure S37.** IR(KBr disc) Spectrum of Compound **4**. **Figure S38.** ^1^HNMR Spectrum of Compound **5** in CDCl_3_. **Figure S39.** ^13^C NMR Spectrum of Compound **5** in CDCl_3_. **Figure S40.** ^1^H-^1^H COSY Spectrum of Compound **5 **in CDCl_3_. **Figure S41.** HSQC Spectrum of Compound **5** in CDCl_3_. **Figure S42.** HMBC Spectrum of Compound **5** in CDCl_3_. **Figure S43.** (-)Total HRESIMS Spectrum of Compound **5**. **Figure S44.** (-) Partial HRESIMS Spectrum of Compound **5**. **Figure S45.** UV Spectrum of Compound **5**. **Figure S46.** IR(KBr disc) Spectrum of Compound **5**. **Figure S47.** ^1^HNMR Spectrum of Compound **6** in CDCl_3_. **Figure S48.** ^13^C NMR Spectrum of Compound **6** in CDCl_3_. **Figure S49.** (+)HRESIMS Spectrum of Compound **6**. **Figure S50.** ^1^HNMR Spectrum of Compound **7** in CDCl_3_. **Figure S51.** ^13^C NMR Spectrum of Compound **7** in CDCl_3_. **Figure S52.** (+)HRESIMS Spectrum of Compound **7**. **Figure S53.** ^1^HNMR Spectrum of Compound **8** in MeOH-*d*_4_. **Figure S54.** ^13^C NMR Spectrum of Compound **8** in MeOH-*d*_4_. **Figure S55.** ^1^H-^1^HCOSY Spectrum of Compound **8** in MeOH-*d*_4_. **Figure S56.** HSQCSpectrum of Compound **8** in MeOH-*d*_4_. **Figure S57.** HMBC Spectrum of Compound **8** in MeOH-*d*_4_. **Figure S58.** (+)HRESIMS Spectrum of Compound **8**. **Table S3.** ^1^H and ^13^C NMR Data of Compound **8**.
