# Supplementary material for: New polychlorinated bibenzyls from Rhododendron minutiflorum
Source: Nat Prod Bioprospect. 2023 Jan 9;13(1):2. doi: 10.1007/s13659-022-00364-x (PMC9826768; doi:10.1007/s13659-022-00364-x)
Supplement: Supplementary file 1 — Additional file 1: Figure S1. Chemical structures ofcompounds 1-8 isolated from R.minutiflorum Hu.Figure S2. Comparison of HPLC-MS of compound 2 and the EtOH extract from R. minutiflorumHu. Figure S3. Comparison of HPLC-MS of compounds 1-5 and the EtOH extract from R. minutiflorumHu. Table S1. X-rayCrystallographic Data for Compound 1. Table S2. X-ray CrystallographicData for Compound 2. Figure S4. 1HNMR Spectrum of Compound 1 in CDCl3. Figure S5. 13C NMR Spectrum of Compound 1 in CDCl3. Figure S6. HSQCSpectrum of Compound 1 in CDCl3. Figure S7. HMBC Spectrum of Compound 1 in CDCl3. Figure S8. (-)Total HRESIMS Spectrum of Compound 1. Figure S9. (-) Partial HRESIMS Spectrum of Compound 1. Figure S10. UV Spectrum of Compound 1. Figure S11. IR(KBr disc) Spectrum of Compound 1. Figure S12. 1HNMR Spectrum of Compound 2 in CDCl3. Figure S13. 13C NMR Spectrum of Compound 2 in CDCl3. Figure S14. 1H-1H COSY Spectrum of Compound 2 in CDCl3. Figure S15. HSQCS pectrum of Compound 2 in CDCl3. Figure S16. HMBC Spectrum of Compound 2 in CDCl3. Figure S17. (-)Total HRESIMS Spectrum of Compound 2. Figure S18. (-) Partial HRESIMS Spectrum of Compound 2. Figure S19. UVSpectrum of Compound 2. Figure S20. IR (KBr disc) Spectrum of Compound 2. Figure S21. 1H NMR Spectrum of Compound 3 in CDCl3. Figure S22. 13C NMR Spectrum of Compound 3 in CDCl3. Figure S23. HSQCSpectrum of Compound 3 in CDCl3. Figure S24. HMBC Spectrum of Compound 3 in CDCl3. Figure S25. (-)Total HRESIMS Spectrum of Compound 3. Figure S26. (-) Partial HRESIMS Spectrum of Compound 3.Figure S27. UV Spectrum of Compound 3. Figure S28. IR(KBr disc) Spectrum of Compound 3. Figure S29. 1H NMR Spectrum of Compound 4 in CDCl3. Figure S30. 13C NMR Spectrum of Compound 4 in CDCl3. Figure S31. 1H-1H COSY Spectrum of Compound 4 in CDCl3. Figure S32. HSQCSpectrum of Compound 4 in CDCl3. Figure S33. HMBC Spectrum of Compound 4 in CDCl3. Figure S34. (-)Total HRESIMS Spectrum of Compound 4. Figure S35. (-) Partial HRESIMS Spectrum of C [file 13659_2022_364_MOESM1_ESM.docx]

**New Polychlorinated Bibenzyls from *Rhododendron minutiflorum* Hu**

Yang-Li Zhu^1#^, Li Deng^1#^, Yu Tang^1^, Xian-Zhe Fan^1^, Yang Han^2^, Mei Pan^3^,

Li-Jun Zhang^1*^ and Hai-Bing Liao^1*^

^1^*State Key Laboratory for Chemistry and Molecular Engineering of Medicinal Resources, Collaborative Innovation Center for Guangxi Ethnic Medicine, School of Chemistry and Pharmaceutical Sciences, Guangxi Normal University, Guilin 541004, People’s Republic of China*

^2^ *Guangxi Key Laboratory of Citrus Biology, Guangxi Academy of Specialty Crops, Guilin 541004, People’s Republic of China*

^3^ *Guilin Pharma Company*, *Guilin 541007, People’s Republic of China*

*Correspondence:* *ajun840618@mailbox.gxnu.edu.cn (L.-J. Zhang); lhbing188@163.com (H.-B. Liao)*

**Contents of Additional Information**

| **NO** | **Content** | **Page** |
| --- | --- | --- |
| Figure S1 | Chemical structures of compounds **1**-**8** isolated from *R. minutiflorum* Hu | 4 |
| Figure S2 | Comparison of HPLC-MS of compound **2** and the EtOH extract from *R. minutiflorum* Hu | 5 |
| Figure S3 | Comparison of HPLC-MS of compounds **1**-**5** and the EtOH extract from *R. minutiflorum* Hu | 6 |
| Table S1 | X-ray Crystallographic Data for Compound **1** | 7 |
| Table S2 | X-ray Crystallographic Data for Compound **2** | 8 |
| Figure S4 | ^1^H NMR Spectrum of Compound **1** in CDCl_3_ | 9 |
| Figure S5 | ^13^C NMR Spectrum of Compound **1** in CDCl_3_ | 10 |
| Figure S6 | HSQC Spectrum of Compound **1** in CDCl_3_ | 11 |
| Figure S7 | HMBC Spectrum of Compound **1** in CDCl_3_ | 12 |
| Figure S8 | (-) Total HRESIMS Spectrum of Compound **1** | 13 |
| Figure S9 | (-) Partial HRESIMS Spectrum of Compound **1** | 14 |
| Figure S10 | UV Spectrum of Compound **1** | 15 |
| Figure S11 | IR (KBr disc) Spectrum of Compound **1** | 16 |
| Figure S12 | ^1^H NMR Spectrum of Compound **2** in CDCl_3_ | 17 |
| Figure S13 | ^13^C NMR Spectrum of Compound **2** in CDCl_3_ | 18 |
| Figure S14 | ^1^H-^1^H COSY Spectrum of Compound **2** in CDCl_3_ | 19 |
| Figure S15 | HSQC Spectrum of Compound **2** in CDCl_3_ | 20 |
| Figure S16 | HMBC Spectrum of Compound **2** in CDCl_3_ | 21 |
| Figure S17 | (-) Total HRESIMS Spectrum of Compound **2** | 22 |
| Figure S18 | (-) Partial HRESIMS Spectrum of Compound **2** | 23 |
| Figure S19 | UV Spectrum of Compound **2** | 24 |
| Figure S20 | IR (KBr disc) Spectrum of Compound **2** | 25 |
| Figure S21 | ^1^H NMR Spectrum of Compound **3** in CDCl_3_ | 26 |
| Figure S22 | ^13^C NMR Spectrum of Compound **3** in CDCl_3_ | 27 |
| Figure S23 | HSQC Spectrum of Compound **3** in CDCl_3_ | 28 |
| Figure S24 | HMBC Spectrum of Compound **3** in CDCl_3_ | 29 |
| Figure S25 | (-) Total HRESIMS Spectrum of Compound **3** | 30 |
| Figure S26 | (-) Partial HRESIMS Spectrum of Compound **3** | 31 |
| Figure S27 | UV Spectrum of Compound **3** | 32 |
| Figure S28 | IR (KBr disc) Spectrum of Compound **3** | 33 |
| Figure S29 | ^1^H NMR Spectrum of Compound **4** in CDCl_3_ | 34 |
| Figure S30 | ^13^C NMR Spectrum of Compound **4** in CDCl_3_ | 35 |
| Figure S31 | ^1^H-^1^H COSY Spectrum of Compound **4** in CDCl_3_ | 36 |
| Figure S32 | HSQC Spectrum of Compound **4** in CDCl_3_ | 37 |
| Figure S33 | HMBC Spectrum of Compound **4** in CDCl_3_ | 38 |
| Figure S34 | (-) Total HRESIMS Spectrum of Compound **4** | 39 |
| Figure S35 | (-) Partial HRESIMS Spectrum of Compound **4** | 40 |
| Figure S36 | UV Spectrum of Compound **4** | 41 |
| Figure S37 | IR (KBr disc) Spectrum of Compound **4** | 42 |
| Figure S38 | ^1^H NMR Spectrum of Compound **5** in CDCl_3_ | 43 |
| Figure S39 | ^13^C NMR Spectrum of Compound **5** in CDCl_3_ | 44 |
| Figure S40 | ^1^H-^1^H COSY Spectrum of Compound **5** in CDCl_3_ | 45 |
| Figure S41 | HSQC Spectrum of Compound **5** in CDCl_3_ | 46 |
| Figure S42 | HMBC Spectrum of Compound **5** in CDCl_3_ | 47 |
| Figure S43 | (-) Total HRESIMS Spectrum of Compound **5** | 48 |
| Figure S44 | (-) Partial HRESIMS Spectrum of Compound **5** | 49 |
| Figure S45 | UV Spectrum of Compound **5** | 50 |
| Figure S46 | IR (KBr disc) Spectrum of Compound **5** | 51 |
| Figure S47 | ^1^H NMR Spectrum of Compound **6** in CDCl_3_ | 52 |
| Figure S48 | ^13^C NMR Spectrum of Compound**6** in CDCl_3_ | 53 |
| Figure S49 | (+) HRESIMS Spectrum of Compound **6** | 54 |
| Figure S50 | ^1^H NMR Spectrum of Compound **7** in CDCl_3_ | 55 |
| Figure S51 | ^13^C NMR Spectrum of Compound **7** in CDCl_3_ | 56 |
| Figure S52 | (+) HRESIMS Spectrum of Compound **7** | 57 |
| Figure S53 | ^1^H NMR Spectrum of Compound **8** in MeOH-*d*_4_ | 58 |
| Figure S54 | ^13^C NMR Spectrum of Compound **8** in MeOH-*d*_4_ | 59 |
| Figure S55 | ^1^H-^1^H COSY Spectrum of Compound **8** in MeOH-*d*_4_ | 60 |
| Figure S56 | HSQC Spectrum of Compound **8** in MeOH-*d*_4_ | 61 |
| Figure S57 | HMBC Spectrum of Compound **8** in MeOH-*d*_4_ | 62 |
| Figure S58 | (+) HRESIMS Spectrum of Compound **8** | 63 |
| Table S3 | ^1^H and ^13^C NMR Data of Compound **8** | 64 |

Figure S1. Chemical structures of compounds **1-8** isolated from *R. minutiflorum* Hu

Figure S2. Comparison of HPLC-MS of compound **2** and the EtOH extract from *R. minutiflorum* Hu

**
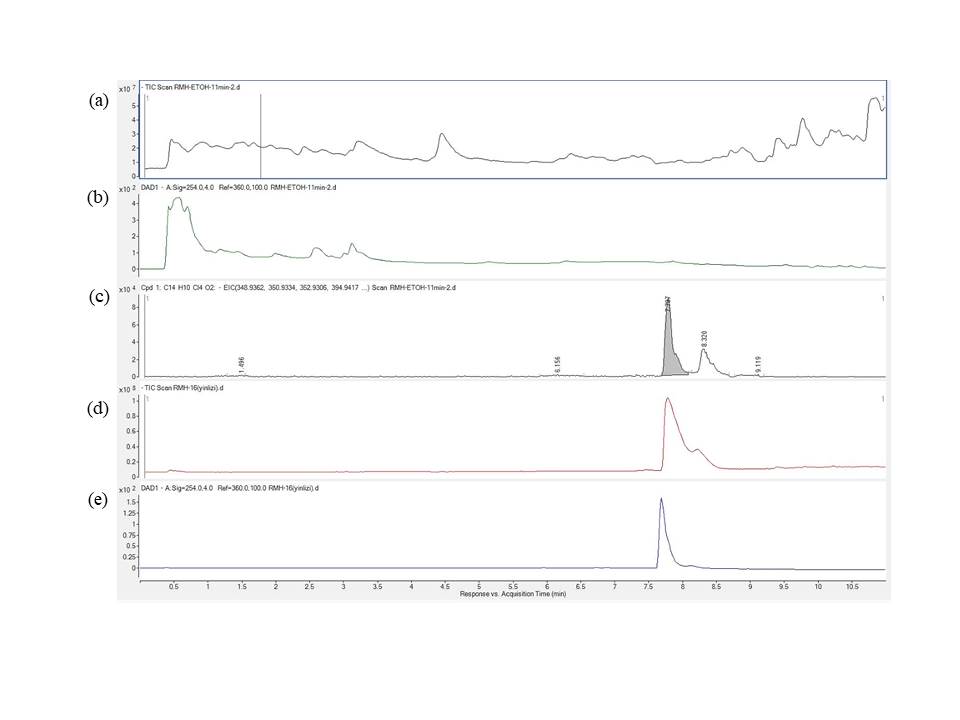
**

Note: (a) The TIC scan of the EtOH extract of *R. minutiflorum* Hu; (b) The HPLC of the EtOH extract of *R. minutiflorum* Hu under 254 nm; (c) The distribution of compound **2** in the TIC scan of the EtOH extract of *R. minutiflorum* Hu; (d) The TIC scan of compound **2**; (e) The HPLC of compound **2** under 254 nm.

Figure S3. Comparison of HPLC-MS of compounds **1**-**5** and the EtOH extract from *R. minutiflorum* Hu

**
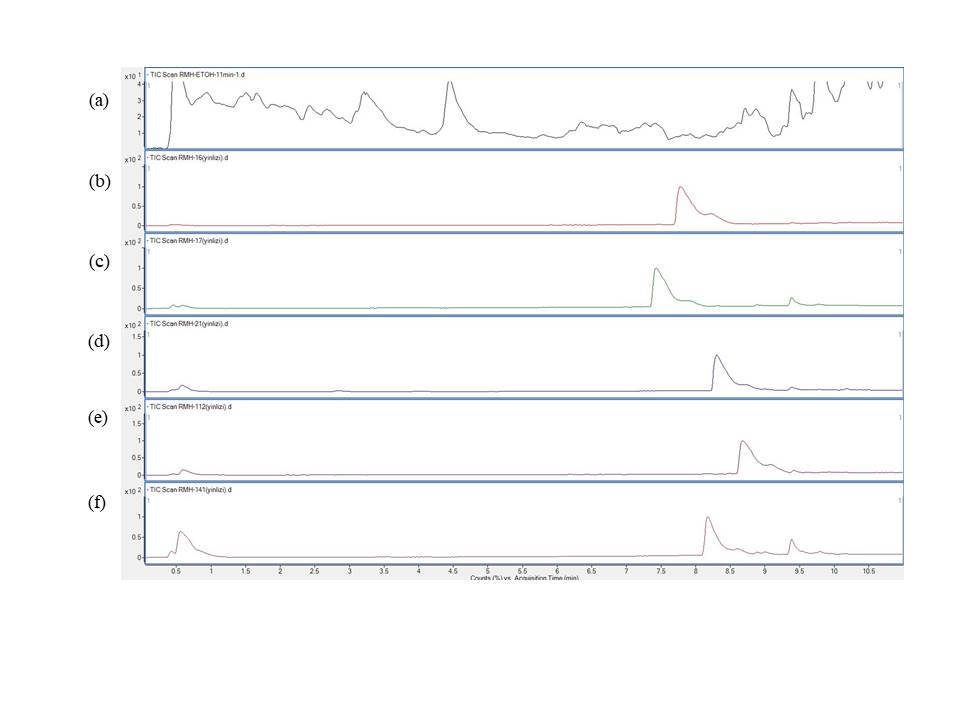
**

Note: (a) The TIC scan of the EtOH extract of *R. minutiflorum* Hu; (b) The TIC scan of compound **2**; (c) The TIC scan of compound **4**; (d) The TIC scan of compound **3**; (e) The TIC scan of compound **1**; (e) The TIC scan of compound **5.**

Table S1. X-ray Crystallographic Data for Compound **1**

| Empirical formula | C_14_H_13_Cl_5_O_4_ |
| --- | --- |
| Moiety formula | C_14_ H_9_ Cl_5_O_2_, 2(H_2_O) |
| Formula weight | 422.49 |
| Temperature/K | 293 |
| Crystal system | monoclinic |
| Space group | I2/a |
| a/Å | 27.1575(11) |
| b/Å | 4.3189(3) |
| c/Å | 30.0170(12) |
| α/° | 90 |
| β/° | 91.182(4) |
| γ/° | 90 |
| Volume/Å^3^ | 3519.9(3) |
| Z | 8 |
| ρ_calc_g/cm^3^ | 1.594 |
| μ/mm^‑1^ | 7.655 |
| F(000) | 1712.0 |
| Crystal size/mm^3^ | 0.1 × 0.05 × 0.03 |
| Radiation | Cu Kα (λ = 1.54184) |
| 2Θ range for data collection/° | 5.89 to 134.15 |
| Index ranges | -32 ≤ h ≤ 32, -4≤ k ≤ 5, -35 ≤ l ≤ 35 |
| Reflections collected | 24050 |
| Independent reflections | 3111 [R_int_ = 0.1191, R_sigma_ = 0.0689] |
| Data/restraints/parameters | 3111/6/222 |
| Goodness-of-fit on F^2^ | 1.125 |
| Final R indexes [I>=2σ (I)] | R_1_ = 0.0710 wR_2_ = 0.1946 |
| Final R indexes [all data] | R_1_ = 0.1132, wR_2_ = 0.2257 |
| Largest diff. peak/hole / e Å^-3^ | 0.64/-0.43 |

Table S2. X-ray Crystallographic Data for Compound **2**

| Empirical formula | C_14_H_10_Cl_4_O_2_ |
| --- | --- |
| Formula weight | 352.02 |
| Temperature/K | 293 |
| Crystal system | orthorhombic |
| Space group | P2_1_2_1_2_1_ |
| a/Å | 4.74049(13) |
| b/Å | 12.6052(5) |
| c/Å | 24.1973(7) |
| α/° | 90 |
| β/° | 90 |
| γ/° | 90 |
| Volume/Å^3^ | 1445.91(8) |
| Z | 4 |
| ρ_calc_g/cm^3^ | 1.617 |
| μ/mm^‑1^ | 7.423 |
| F(000) | 712.0 |
| Crystal size/mm^3^ | 0.15 × 0.03 × 0.03 |
| Radiation | Cu Kα (λ = 1.54184) |
| 2Θ range for data collection/° | 7.306 to 134.126 |
| Index ranges | -5 ≤ h ≤ 3, -15 ≤ k ≤ 14, -26 ≤ l ≤ 28 |
| Reflections collected | 6362 |
| Independent reflections | 2567 [R_int_ = 0.0471, R_sigma_ = 0.0556] |
| Data/restraints/parameters | 2567/0/182 |
| Goodness-of-fit on F^2^ | 0.991 |
| Final R indexes [I>=2σ (I)] | R_1_ = 0.0330, wR_2_ = 0.0744 |
| Final R indexes [all data] | R_1_ = 0.0386, wR_2_ = 0.0768 |
| Largest diff. peak/hole / e Å^-3^ | 0.21/-0.21 |
| Flack parameter | -0.008(14) |

Figure S4. ^1^H NMR Spectrum of Compound **1** in CDCl_3_


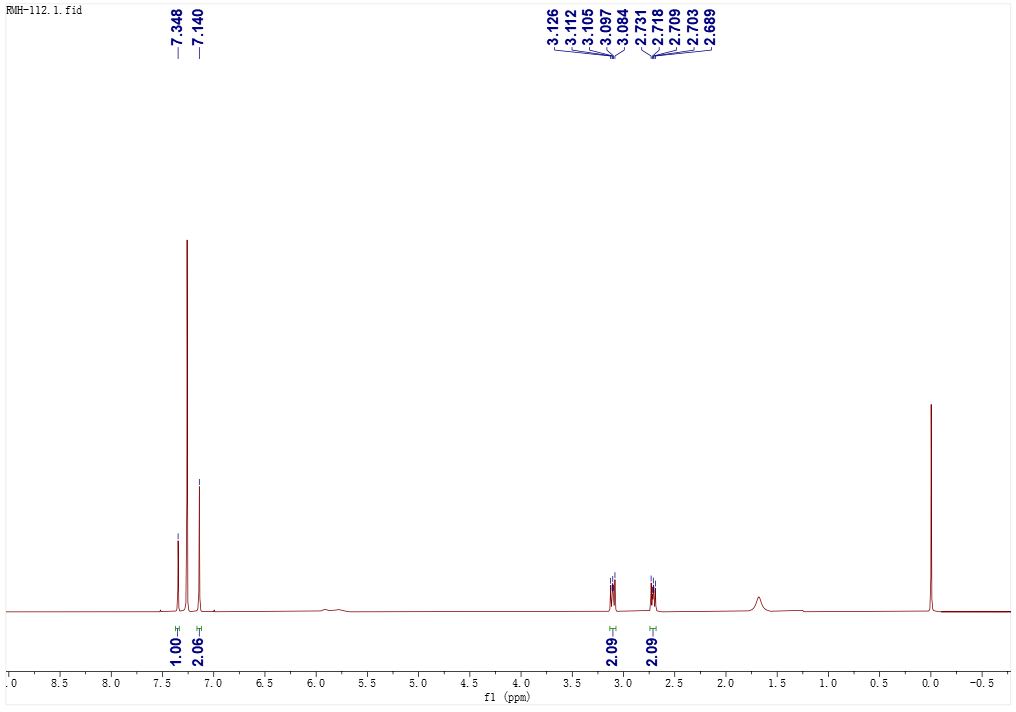


Figure S5. ^13^C NMR Spectrum of Compound **1** in CDCl_3_


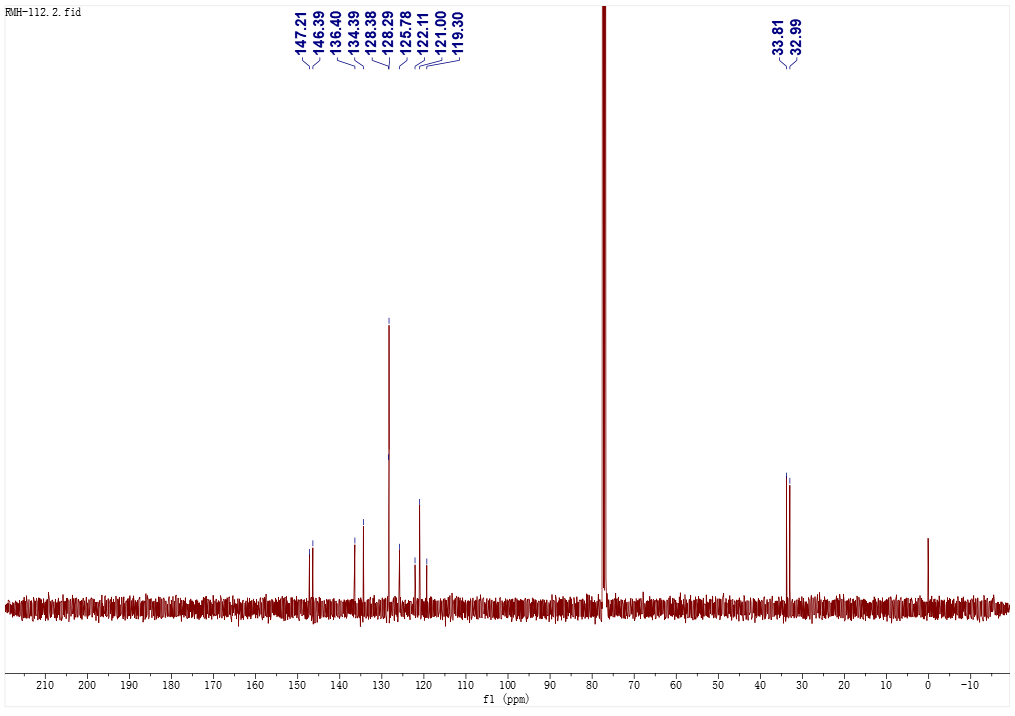


Figure S6. HSQC Spectrum of Compound **1** in CDCl_3_


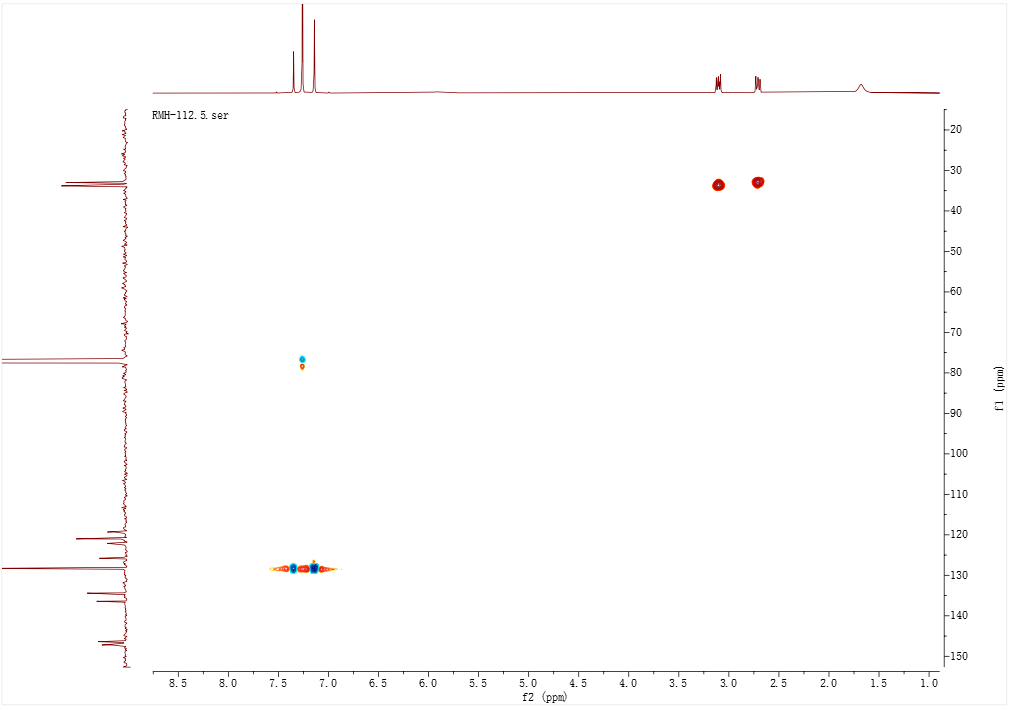


Figure S7. HMBC Spectrum of Compound **1** in CDCl_3_


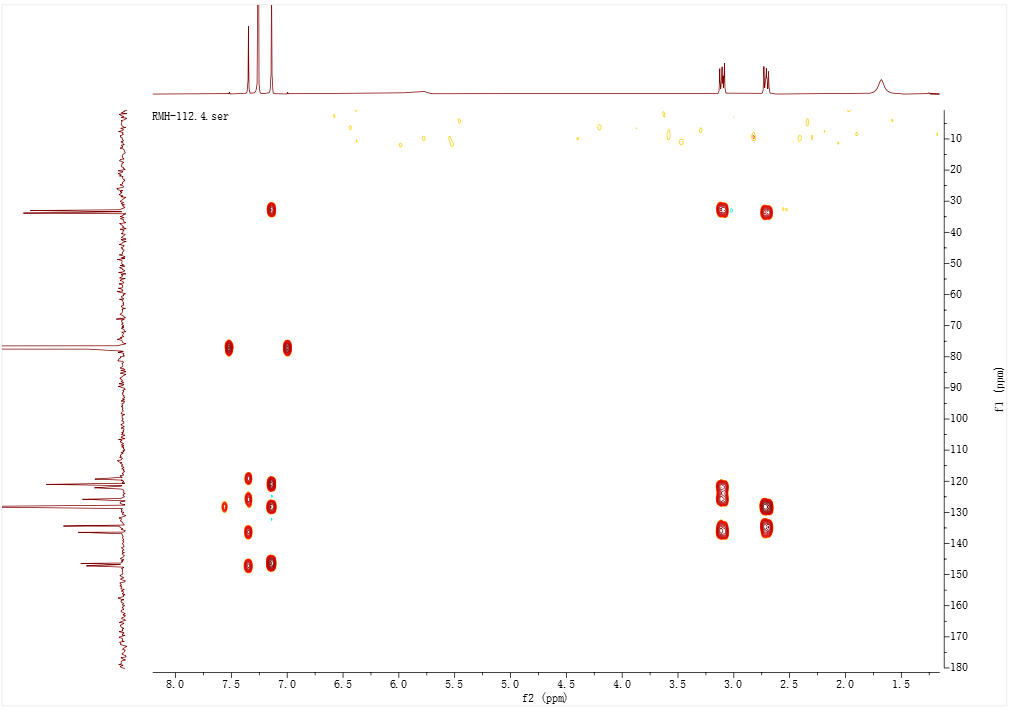


Figure S8. (-) Total HRESIMS Spectrum of Compound **1**

__
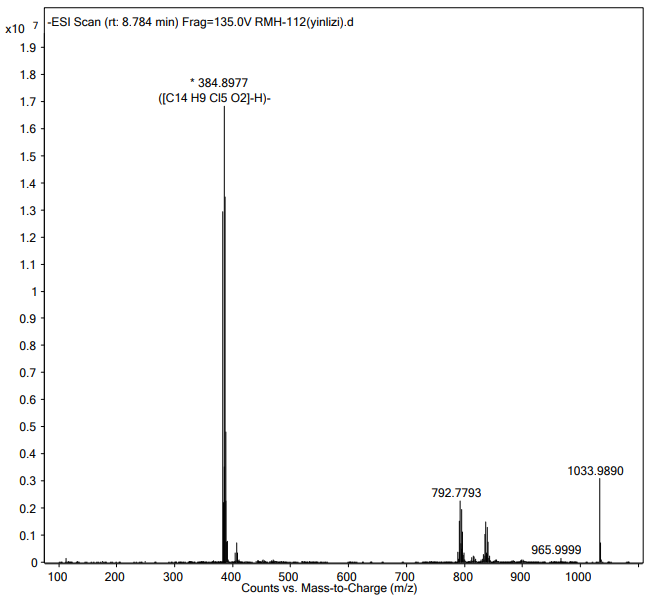


Figure S9 (-) Partial HRESIMS Spectrum of Compound **1**


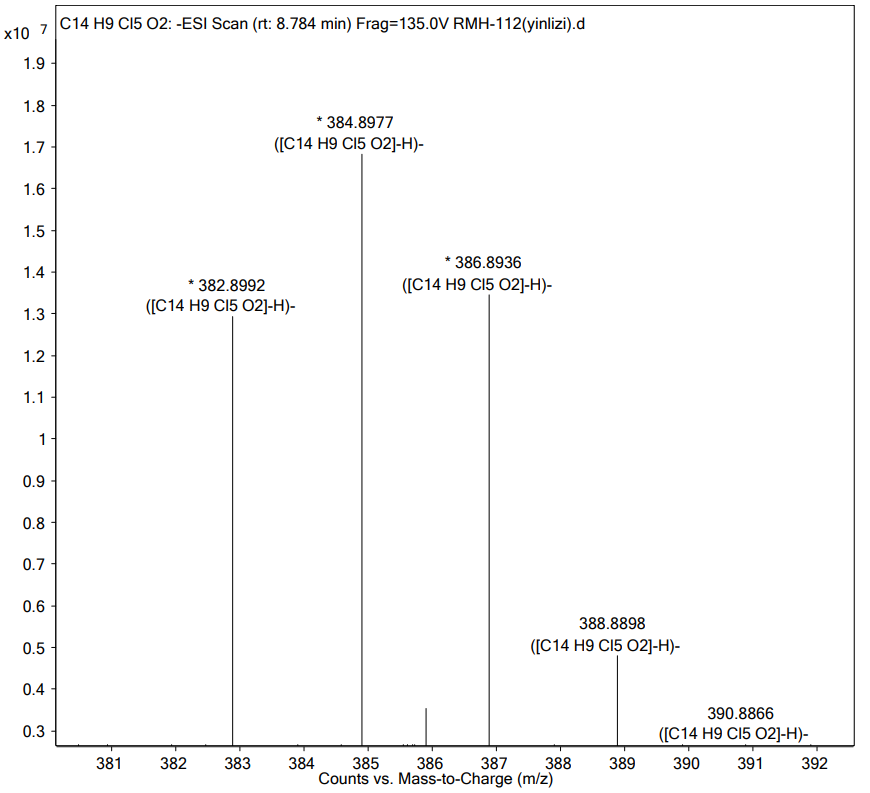


Figure S10. UV Spectrum of Compound **1**

_
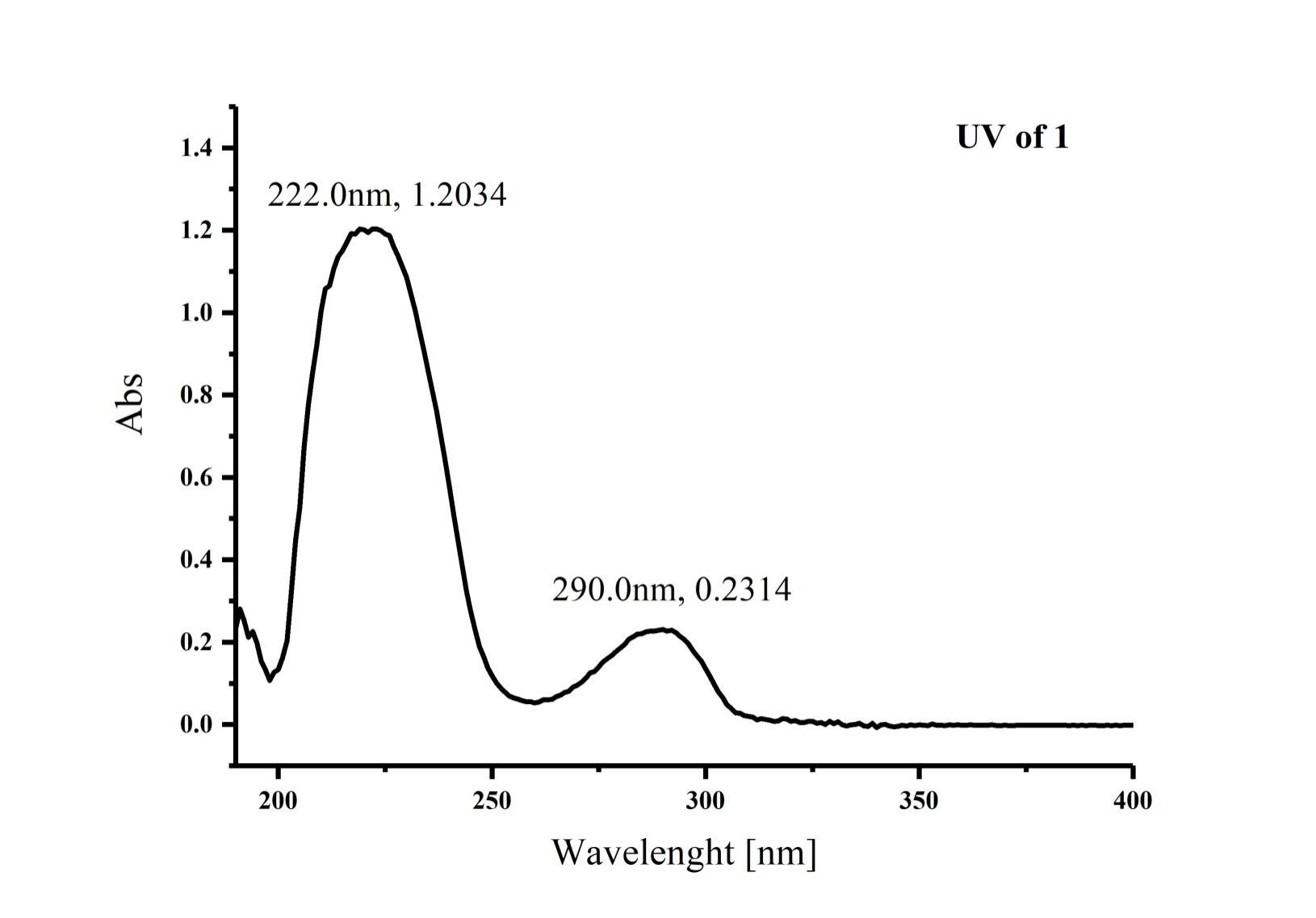
_

Figure S11. IR (KBr disc) Spectrum of Compound **1**

_
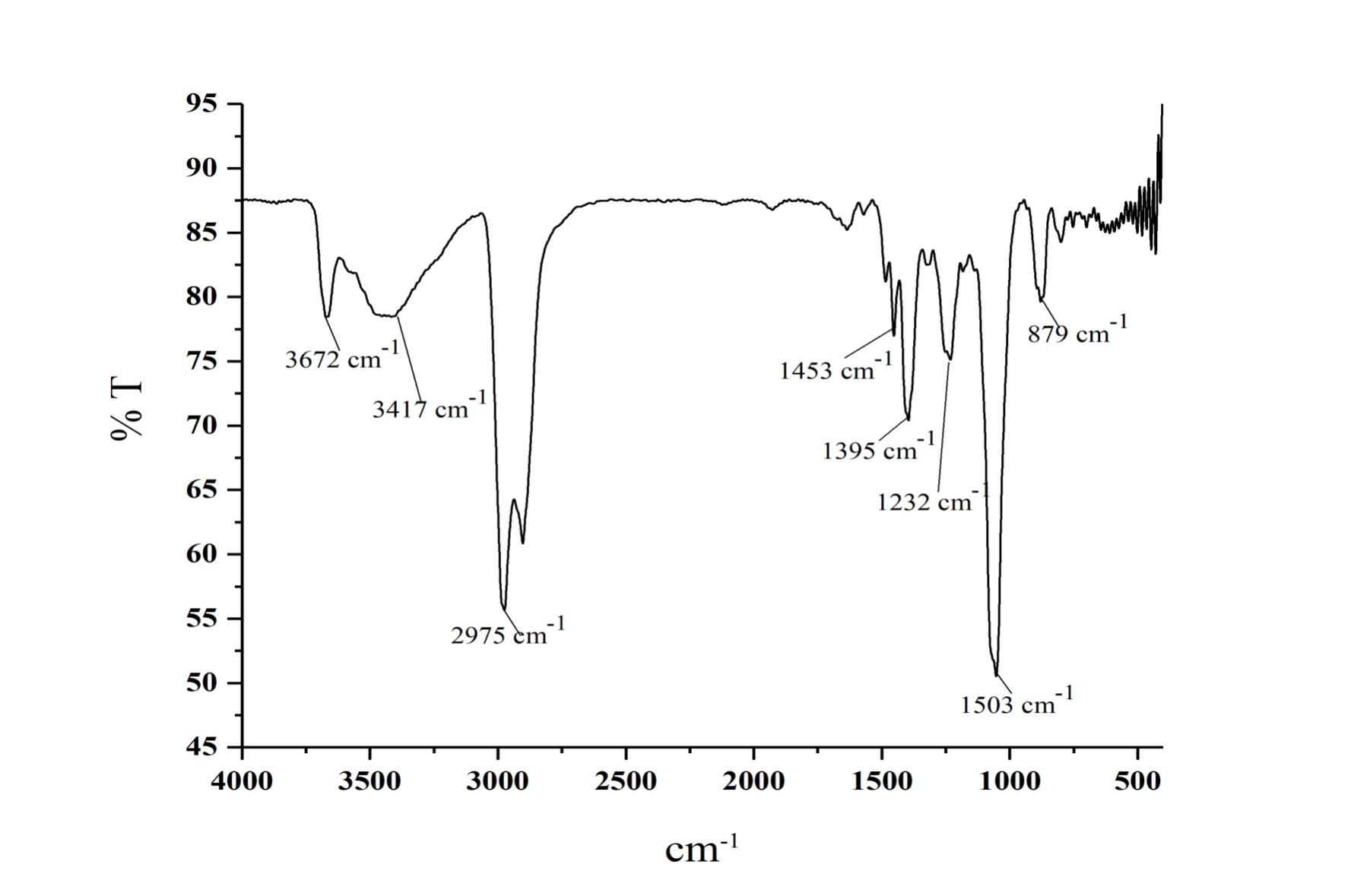
_

Figure S12. ^1^H NMR Spectrum of Compound **2** in CDCl_3_


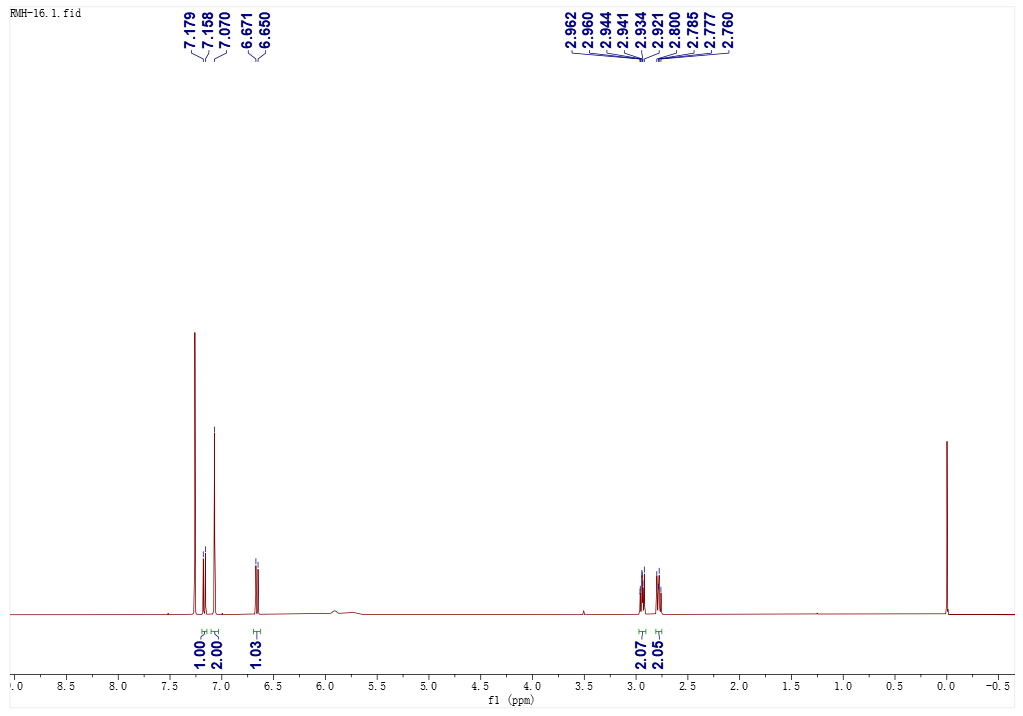


Figure S13. ^13^C NMR Spectrum of Compound **2** in CDCl_3_


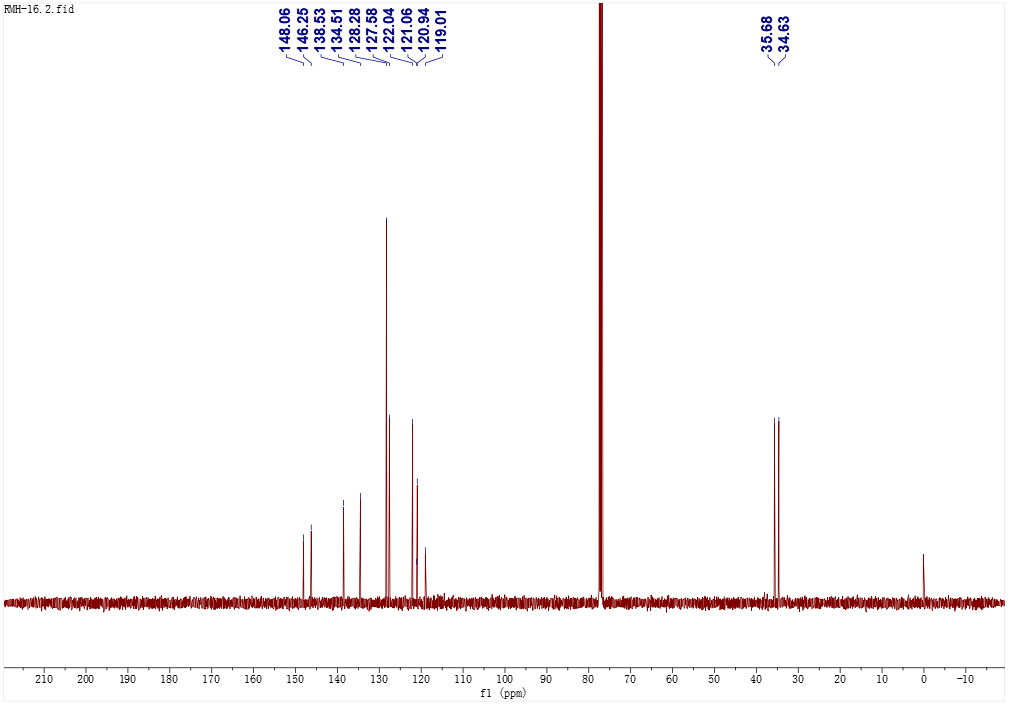


Figure S14. ^1^H-^1^H COSY Spectrum of Compound **2** in CDCl_3_


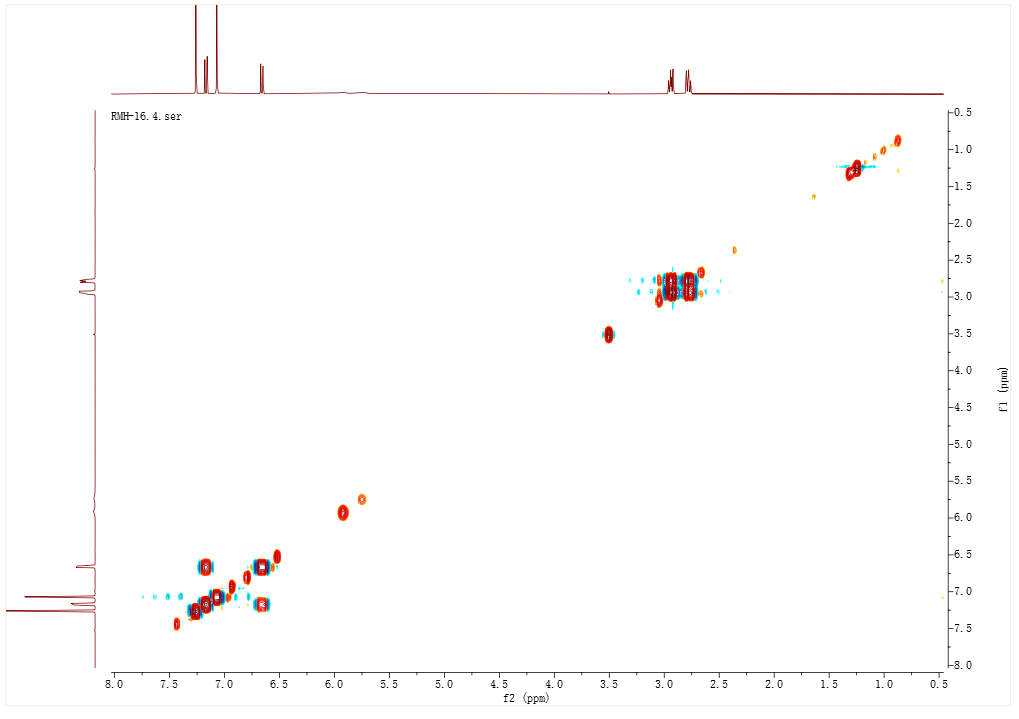


Figure S15. HSQC Spectrum of Compound **2** in CDCl_3_


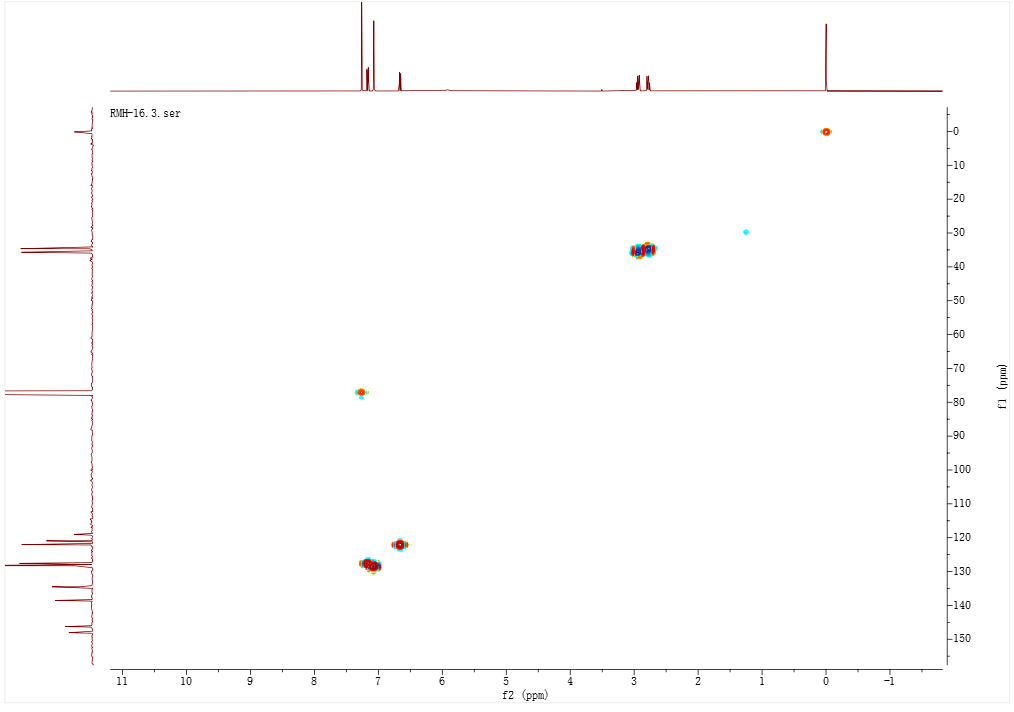


Figure S16.HMBC Spectrum of Compound **2** in CDCl_3_


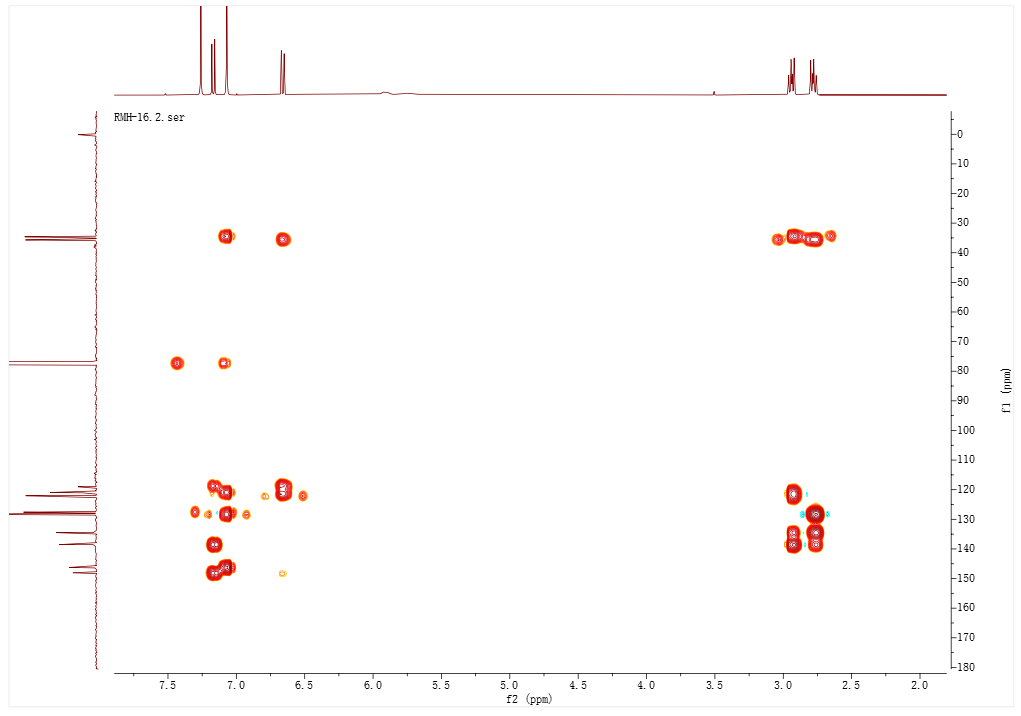


Figure S17. (-) Total HRESIMS Spectrum of Compound **2**


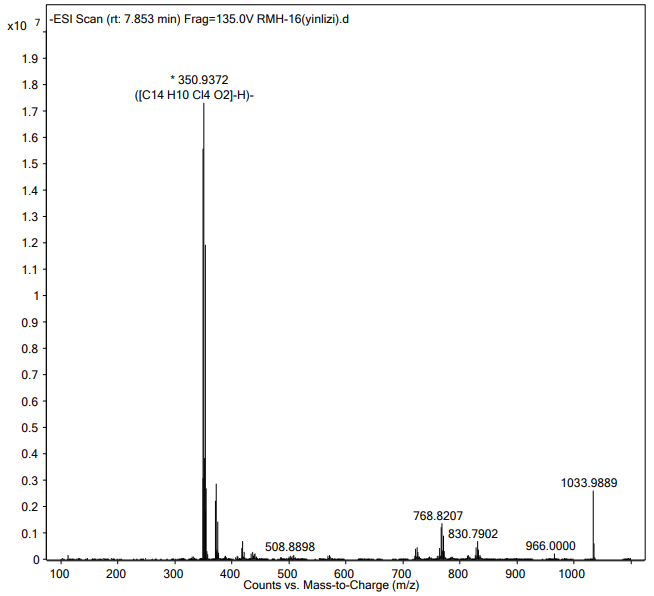


Figure S18. (-) Partial HRESIMS Spectrum of Compound **2**


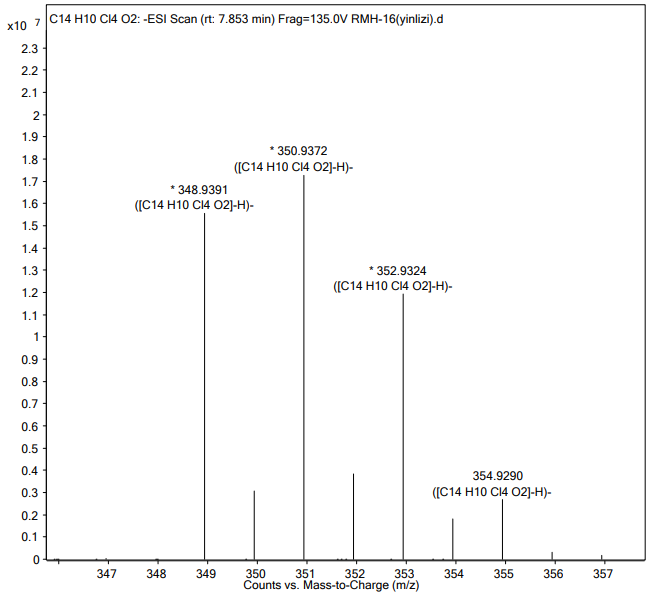


Figure S19. UV Spectrum of Compound **2**


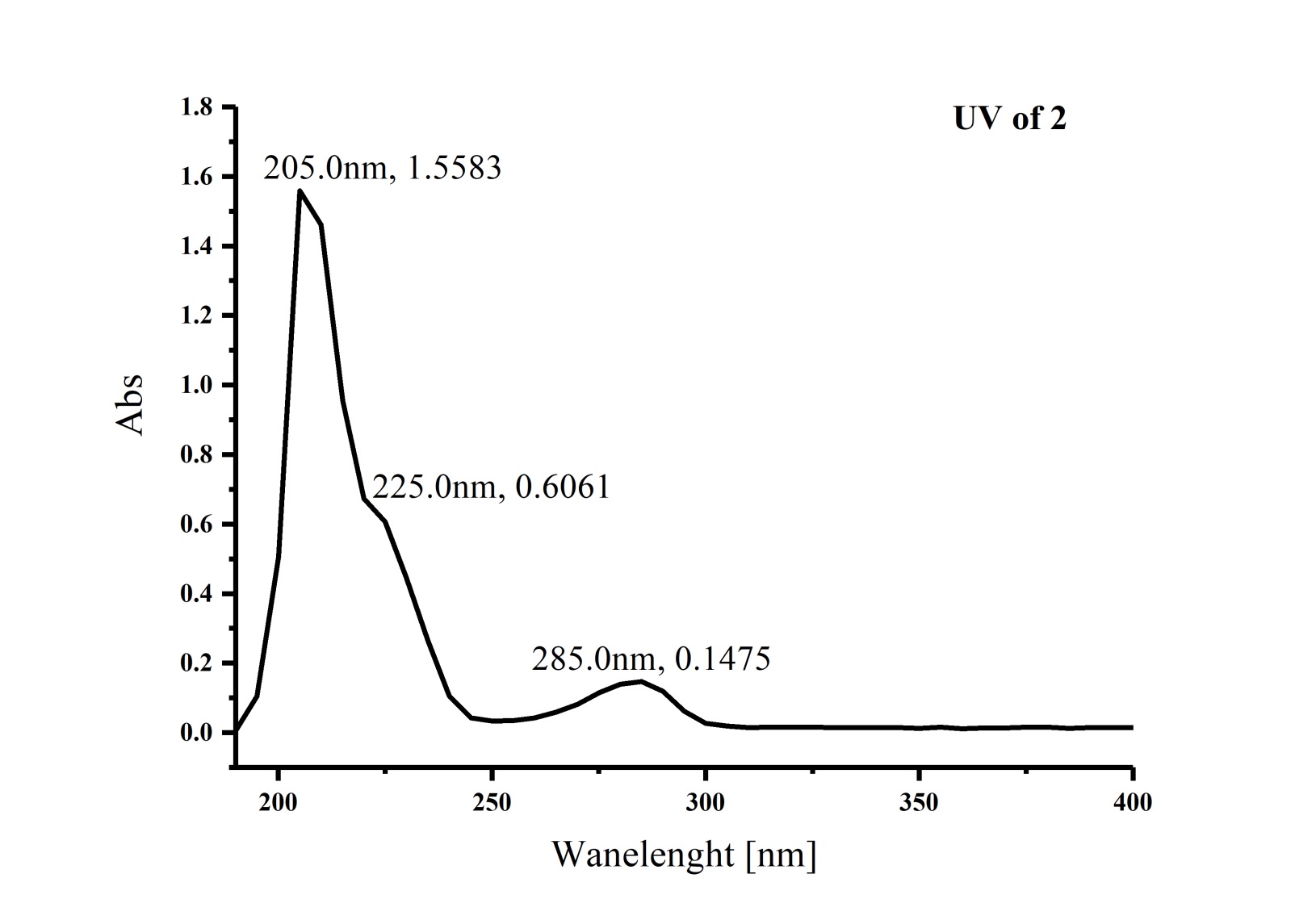


Figure S20. IR (KBr disc) Spectrum of Compound **2**


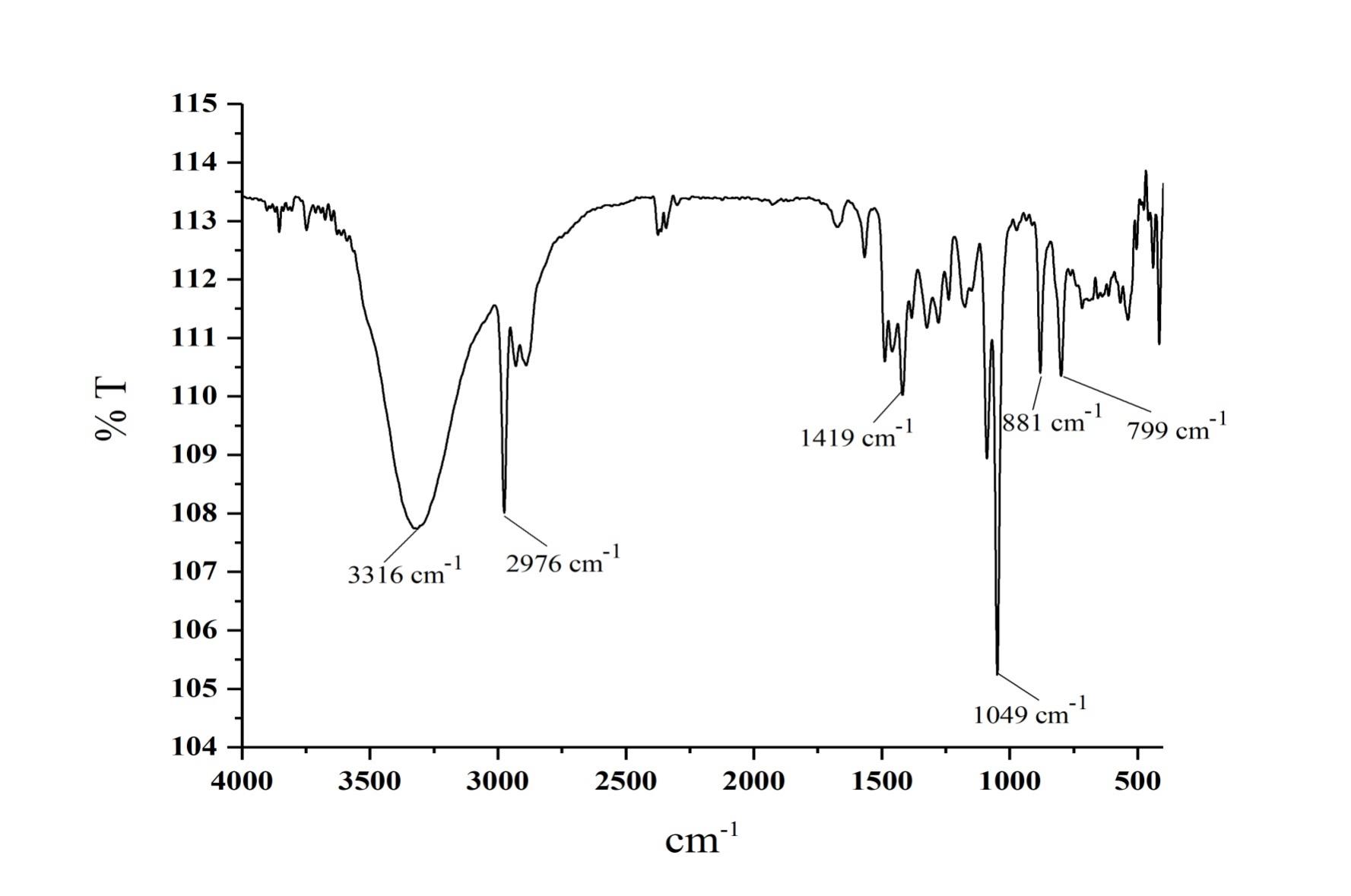


Figure S21. ^1^H NMR Spectrum of Compound **3** in CDCl_3_


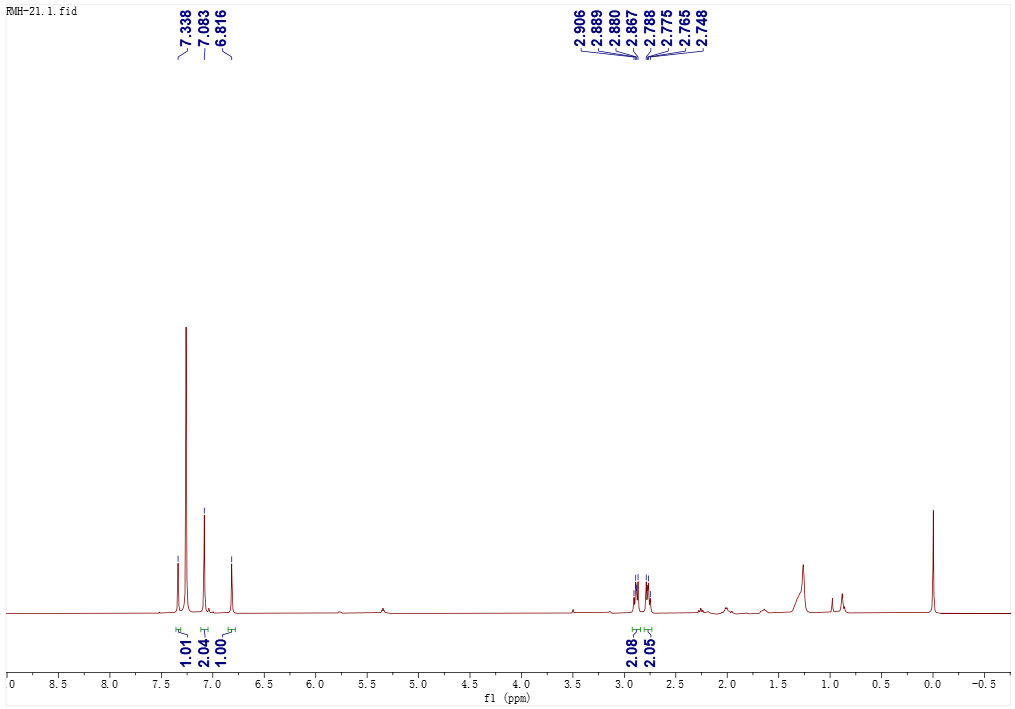


Figure S22. ^13^C NMR Spectrum of Compound **3** in CDCl_3_


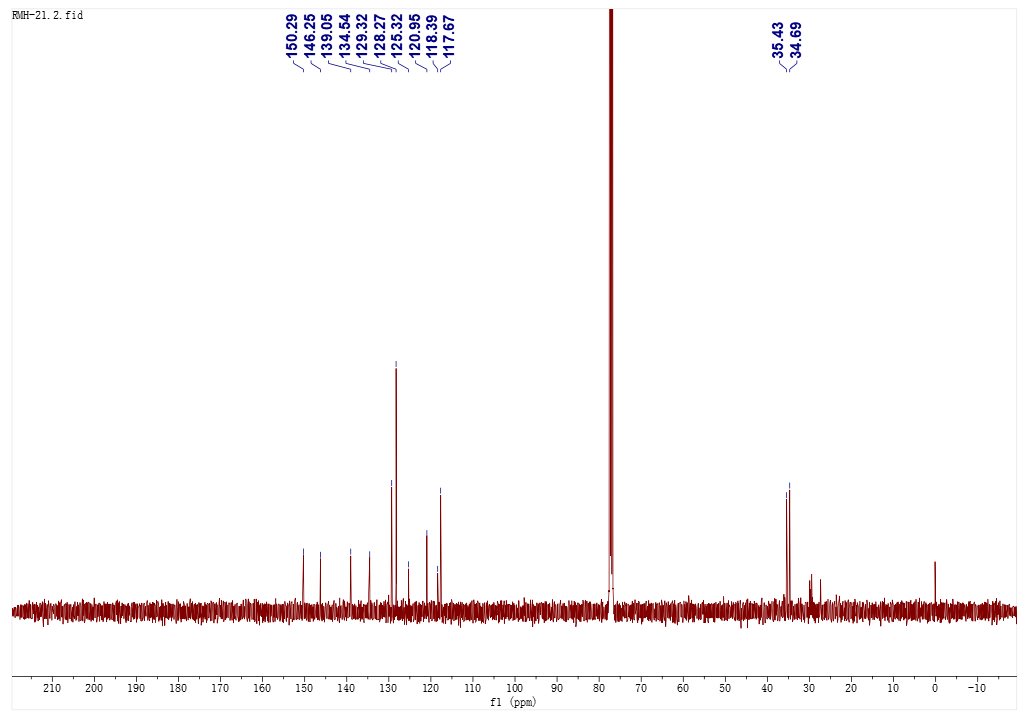


Figure S23. HSQC Spectrum of Compound **3** in CDCl_3_


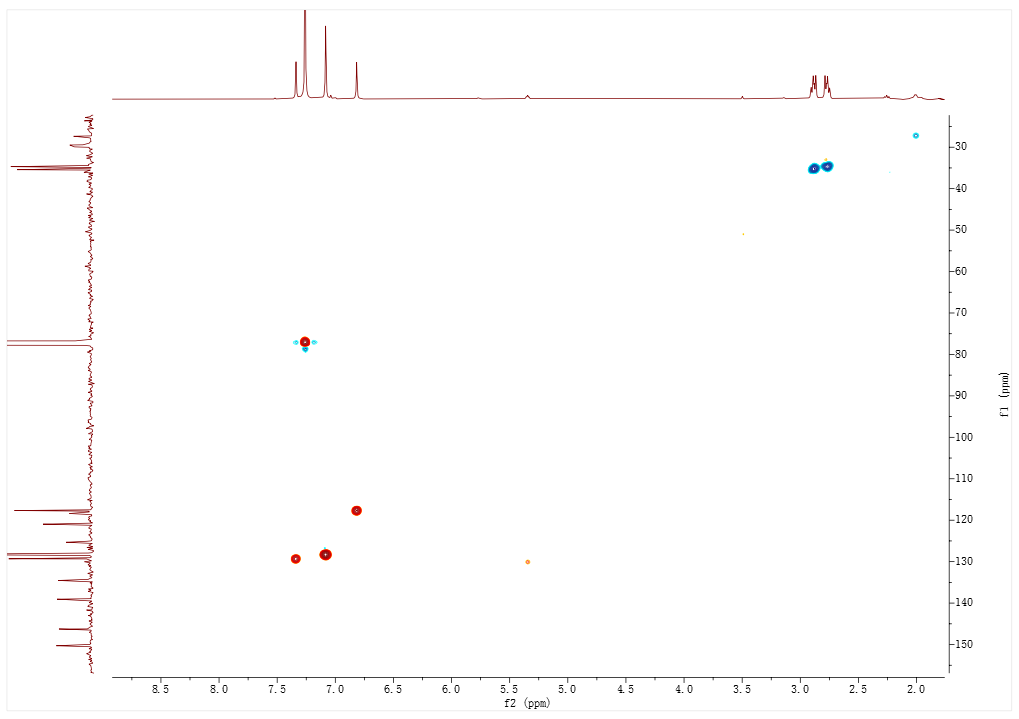


Figure S24. HMBC Spectrum of Compound **3** in CDCl_3_


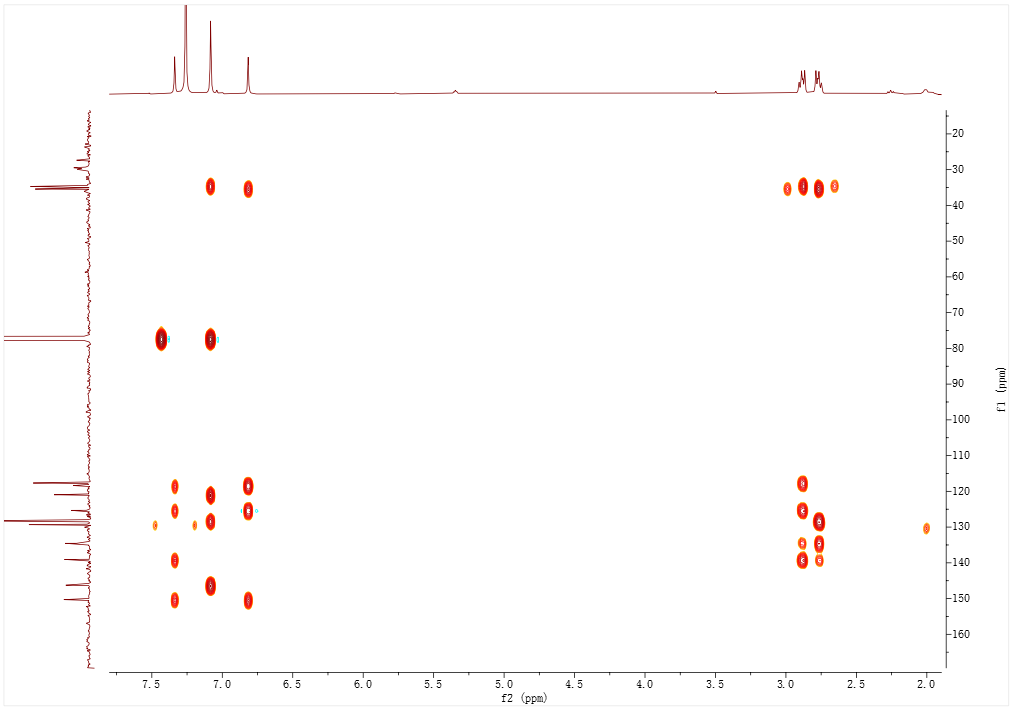


Figure S25. (-) Total HRESIMS Spectrum of Compound **3**


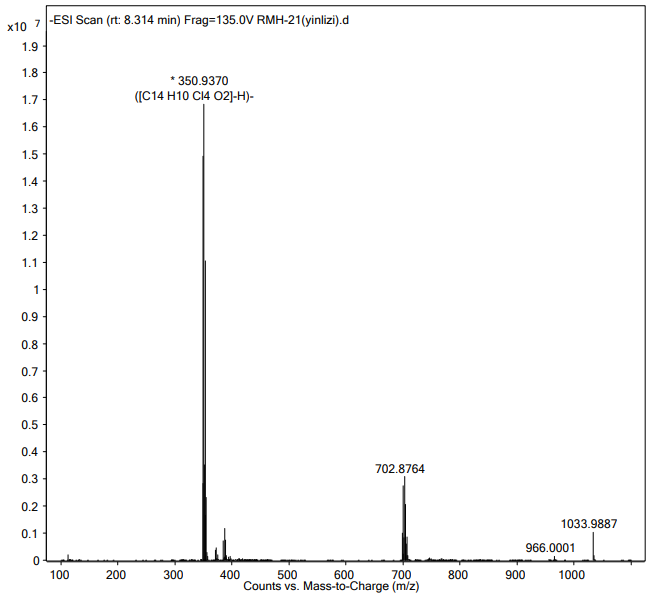


Figure S26. (-) Partial HRESIMS Spectrum of Compound **3**


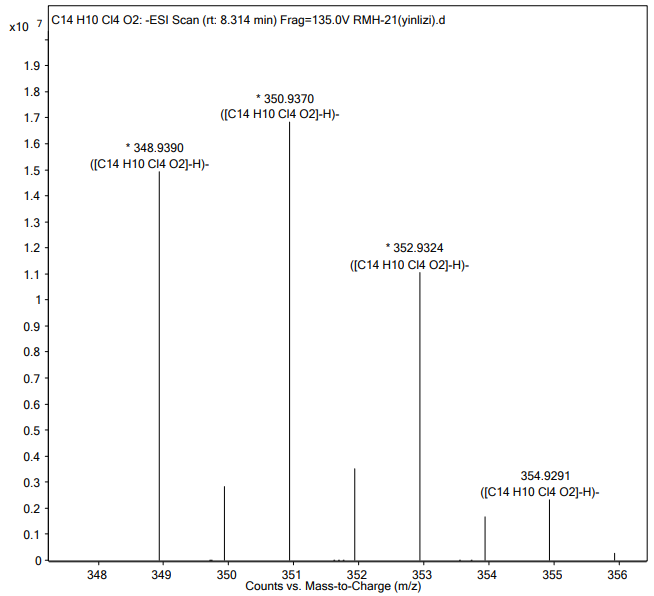


Figure S27. UV Spectrum of Compound **3**


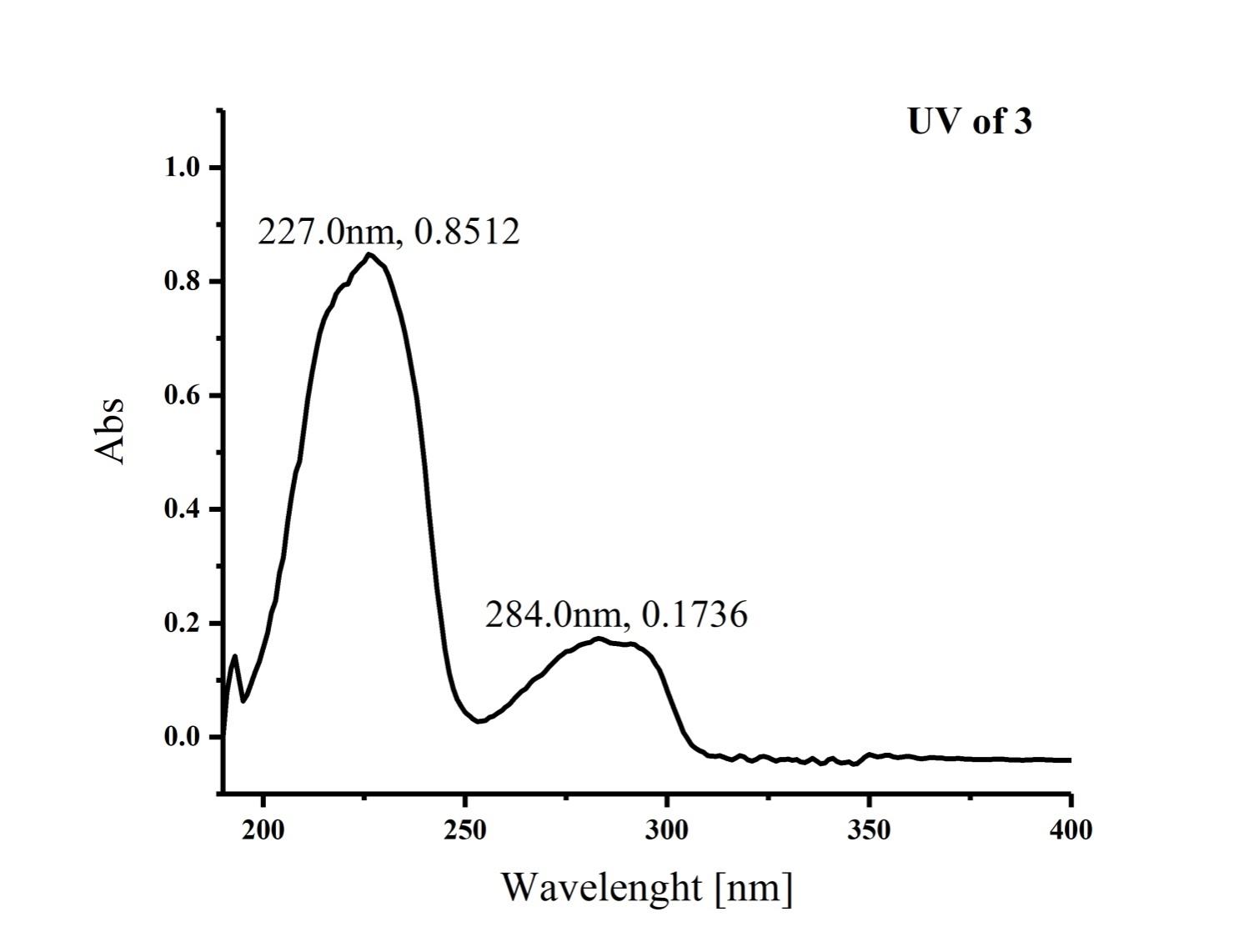


Figure S28. IR (KBr disc) Spectrum of Compound **3**


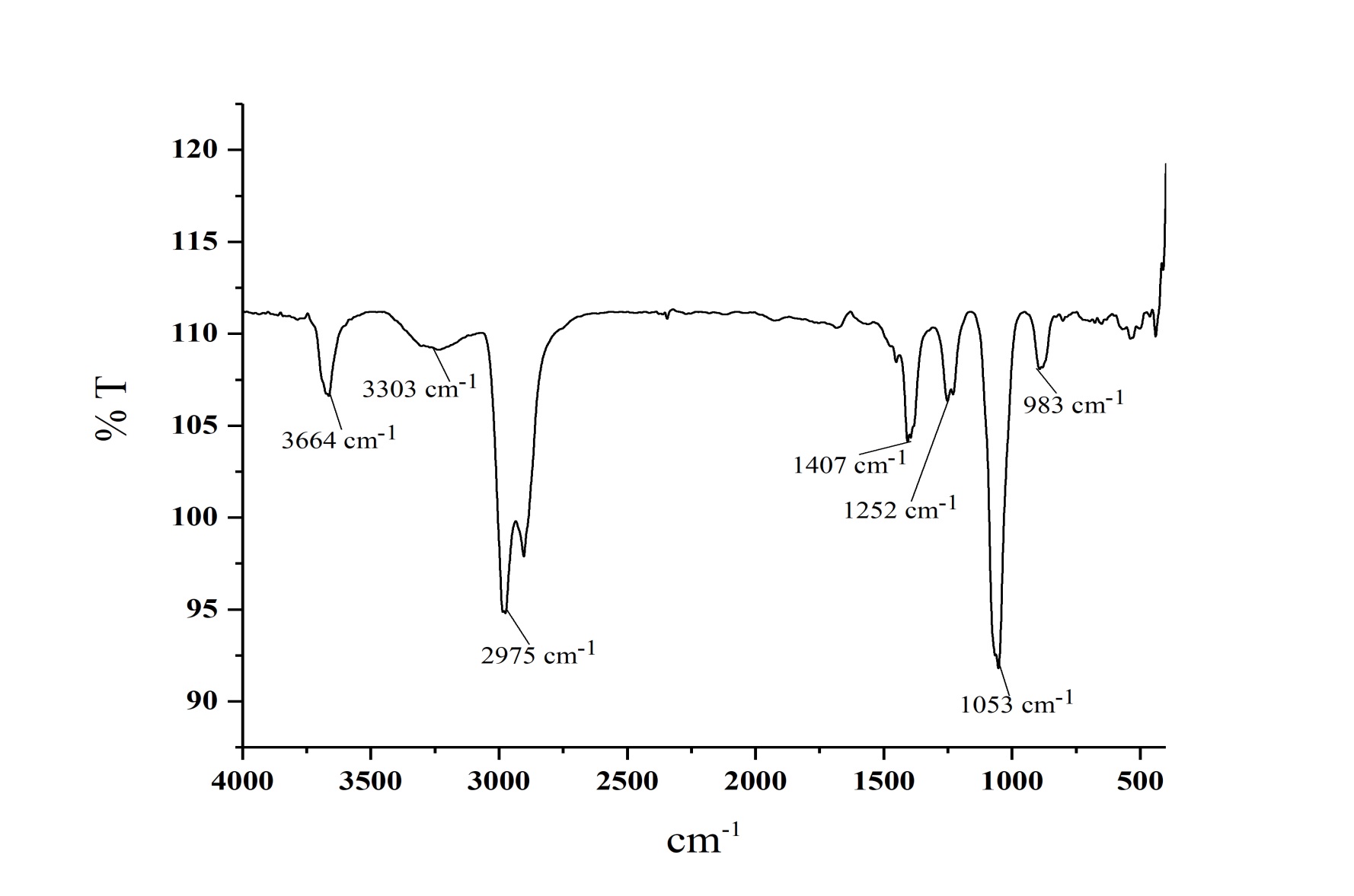


Figure S29. ^1^H NMR Spectrum of Compound **4** in CDCl_3_


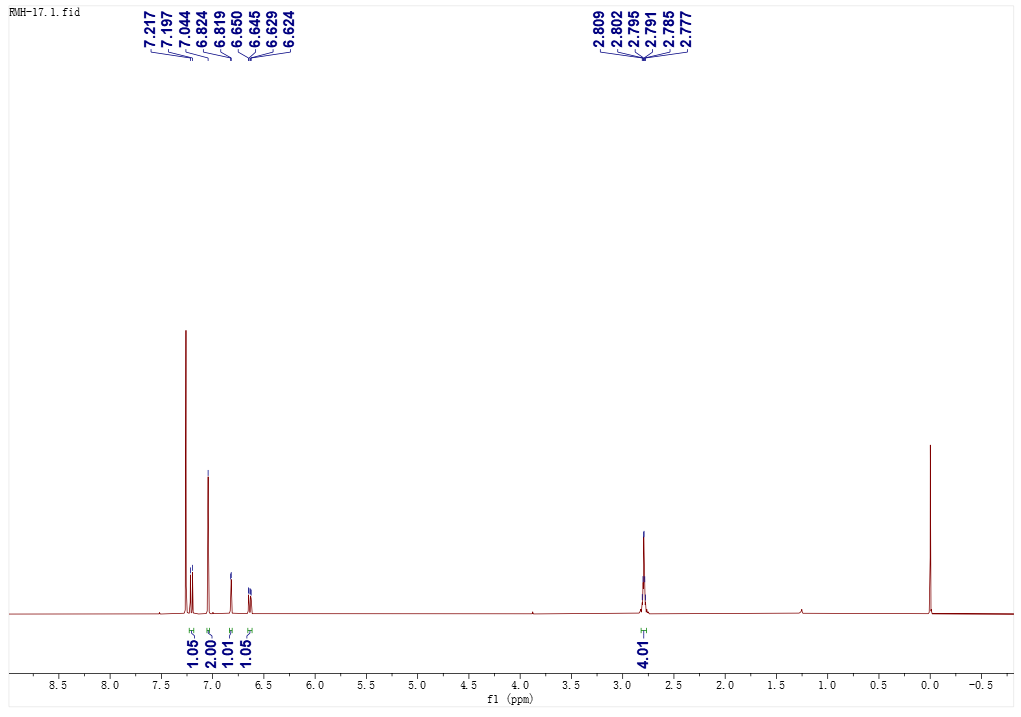


Figure S30. ^13^C NMR Spectrum of Compound **4** in CDCl_3_


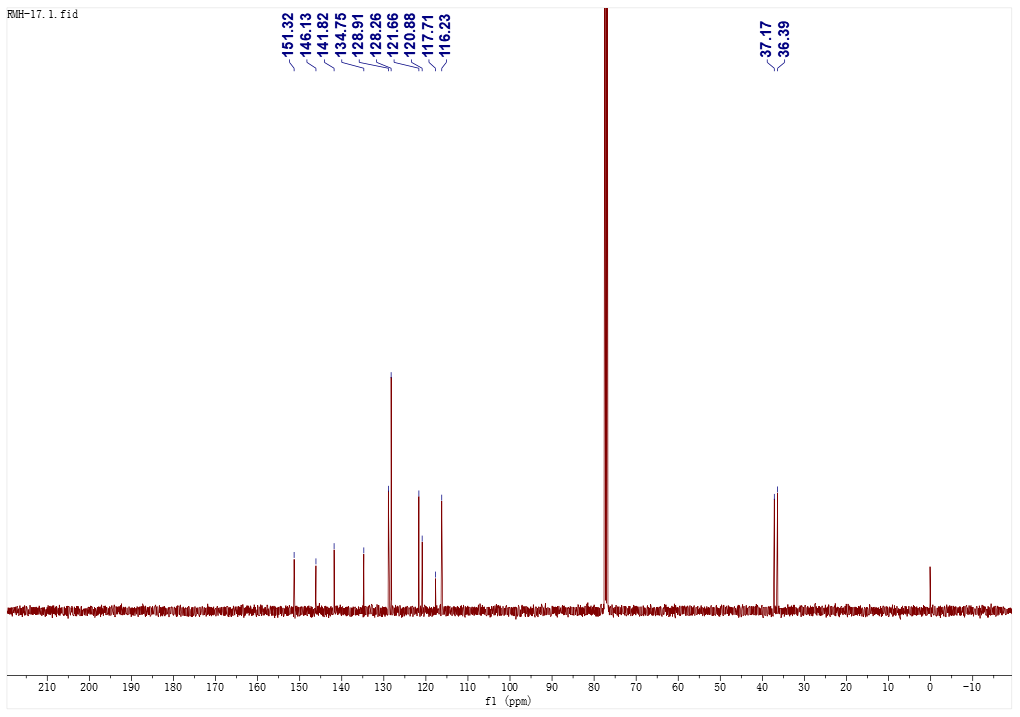


Figure S31. ^1^H-^1^H COSY Spectrum of Compound **4** in CDCl_3_


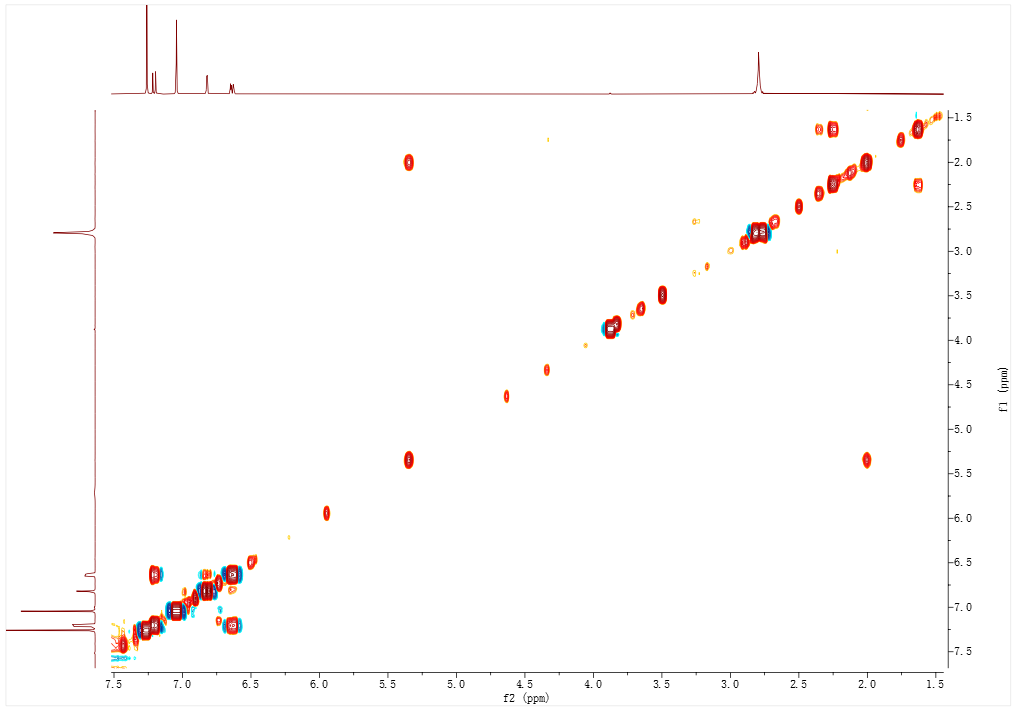


Figure S32. HSQC Spectrum of Compound **4** in CDCl_3_


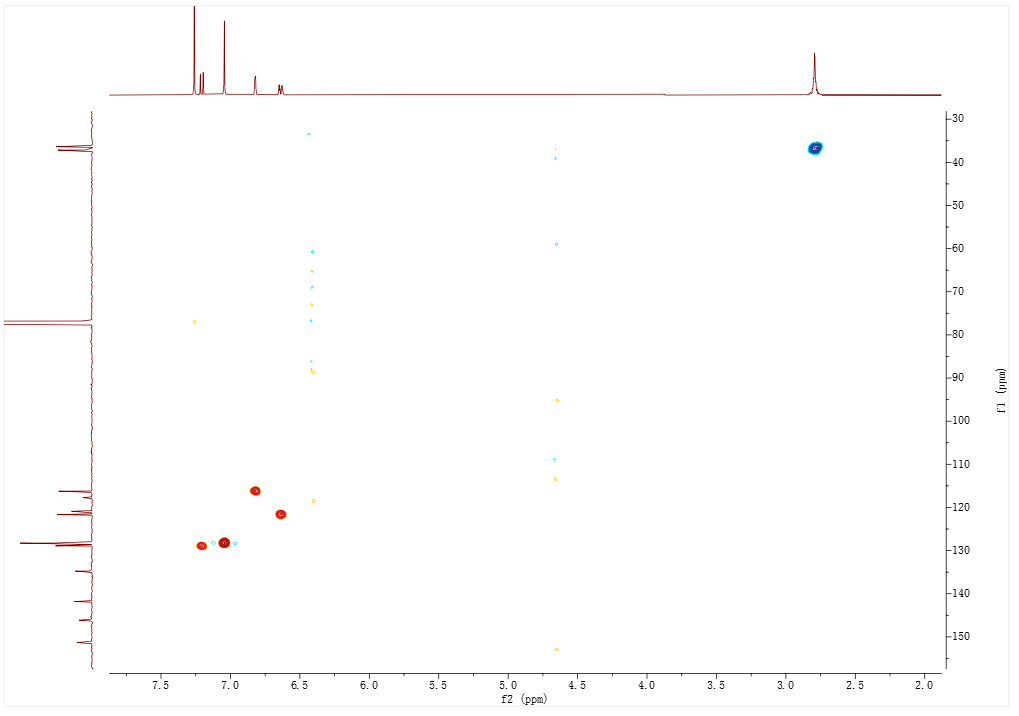


Figure S33. HMBC Spectrum of Compound **4** in CDCl_3_


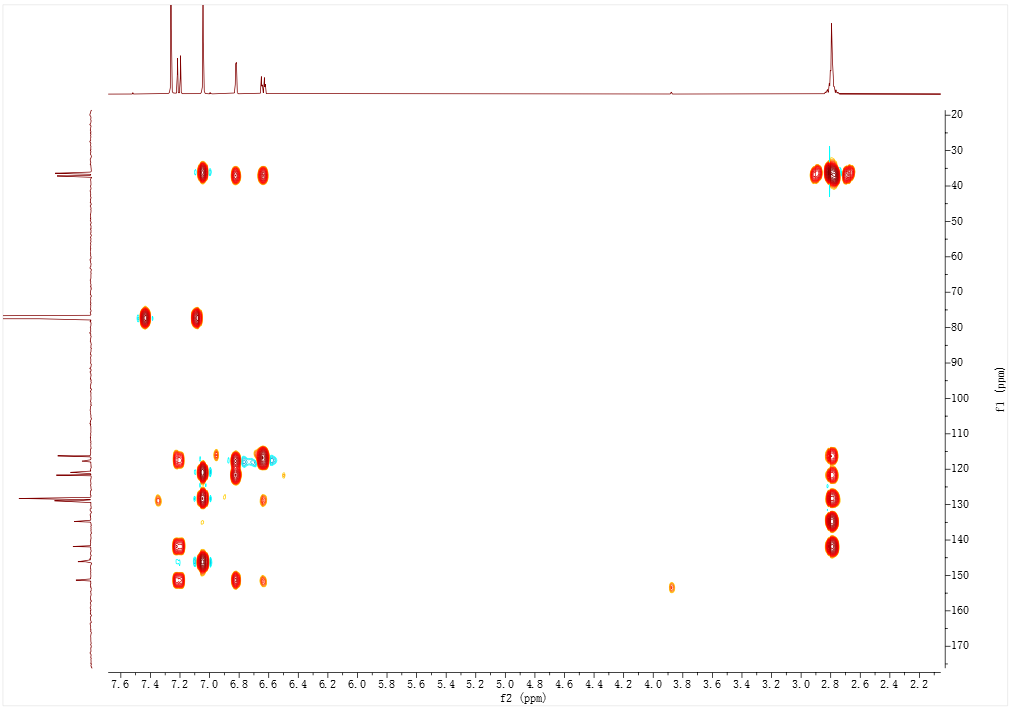


Figure S34. (-) Total HRESIMS Spectrum of Compound **4**


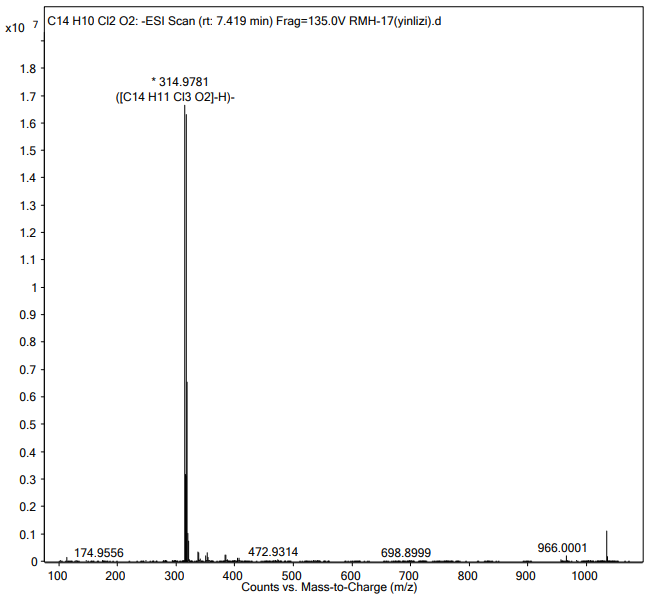


Figure S35. (-) Partial HRESIMS Spectrum of Compound **4**


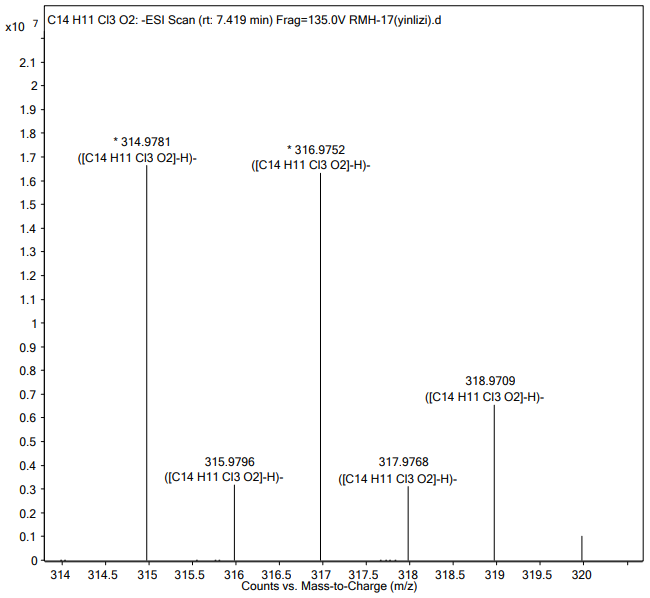


Figure S36. UV Spectrum of Compound **4**


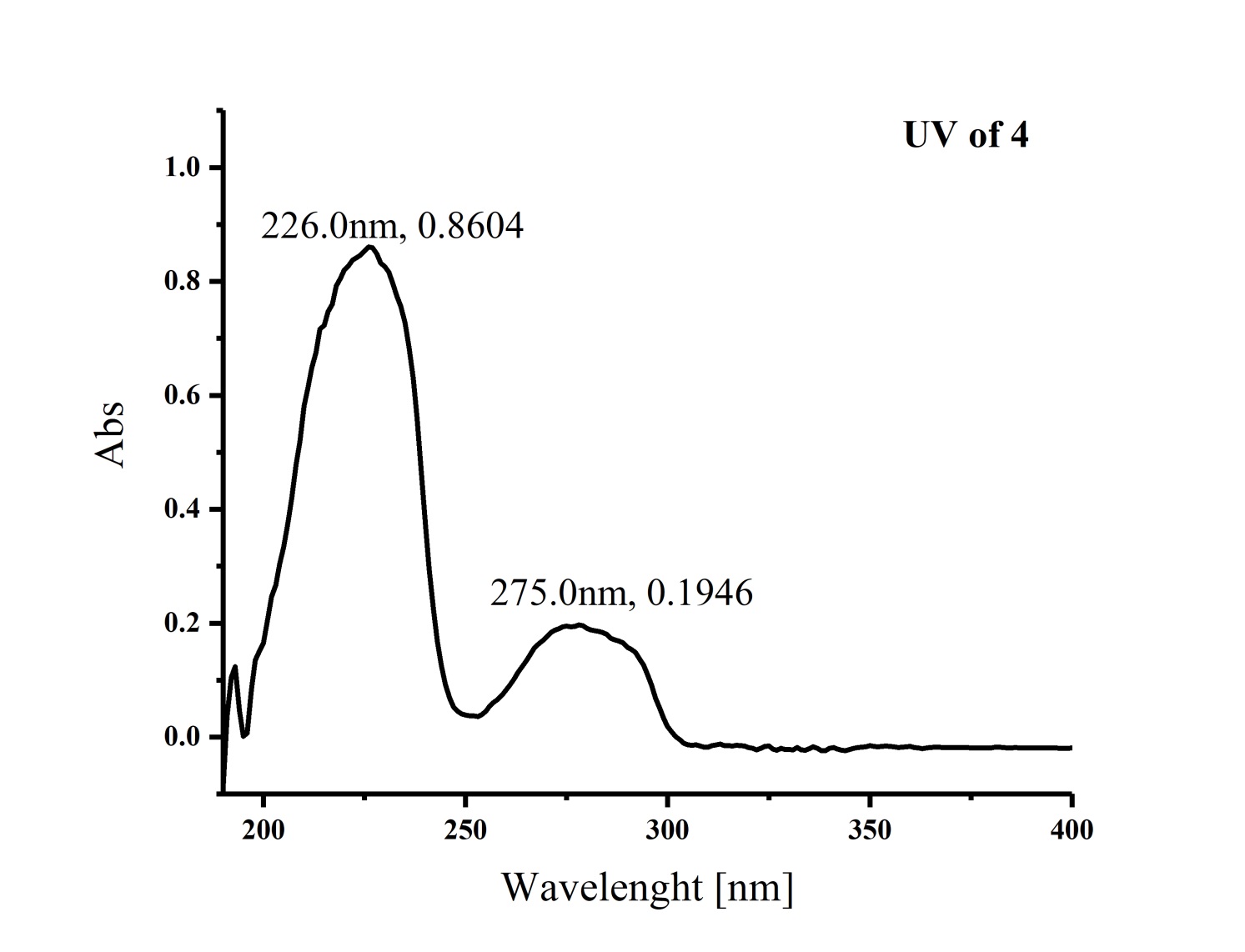


Figure S37. IR (KBr disc) Spectrum of Compound **4**


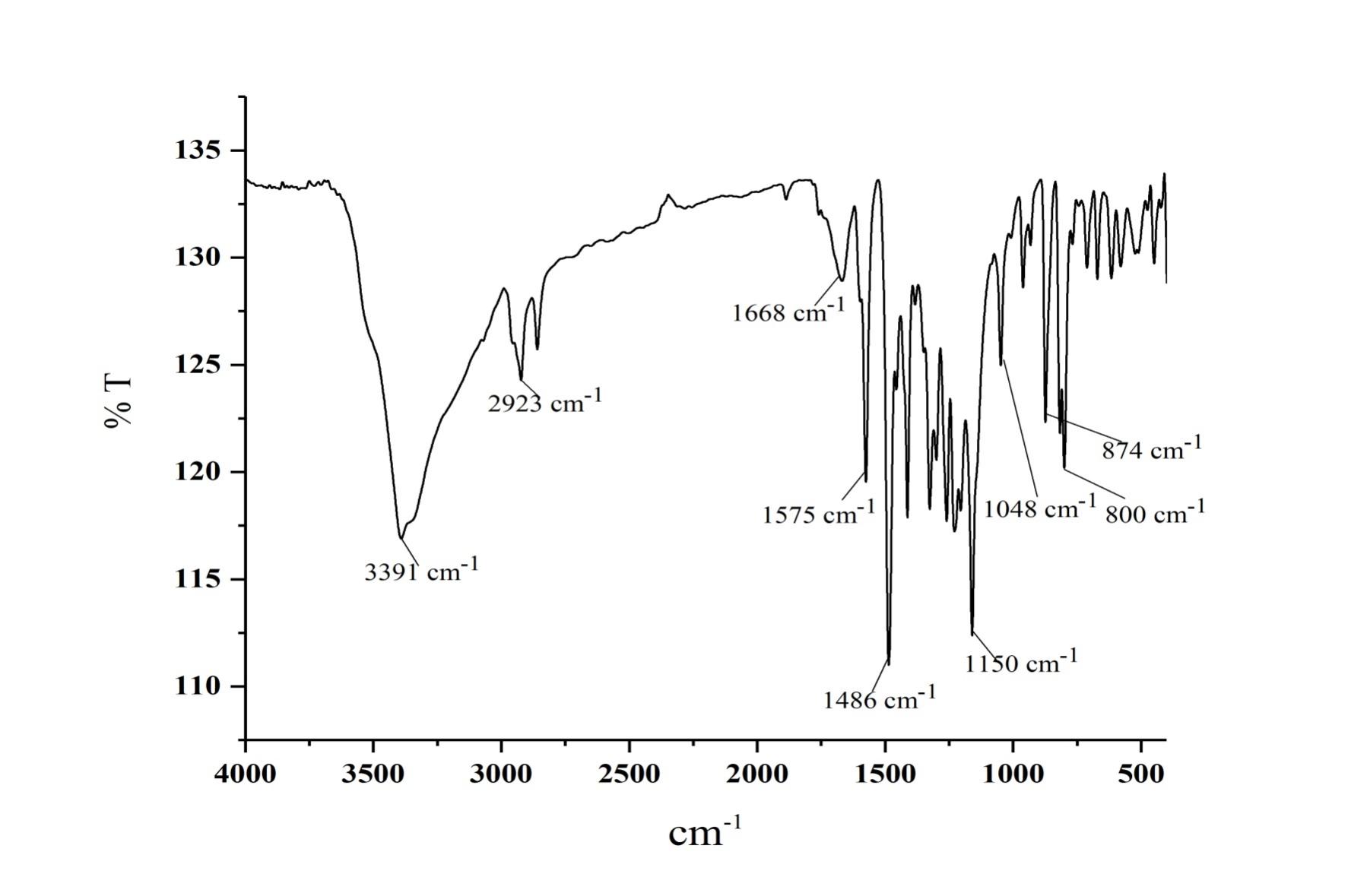


Figure S38. ^1^H NMR Spectrum of Compound **5** CDCl_3_


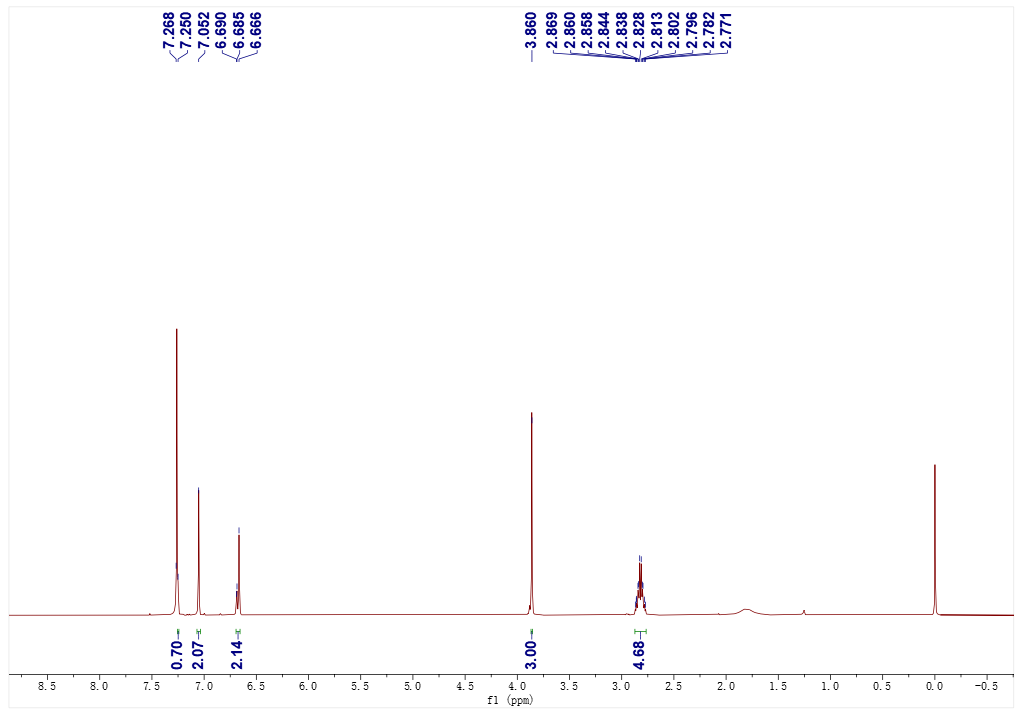


Figure S39. ^13^C NMR Spectrum of Compound **5** in CDCl_3_


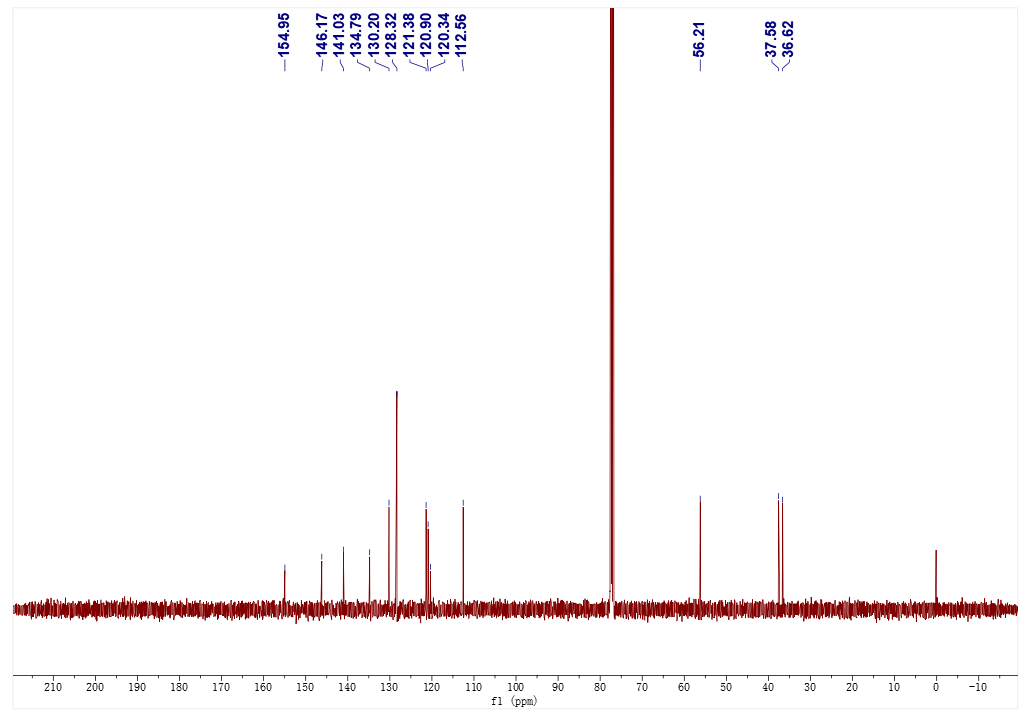


Figure S40. ^1^H-^1^H COSY Spectrum of Compound **5** in CDCl_3_


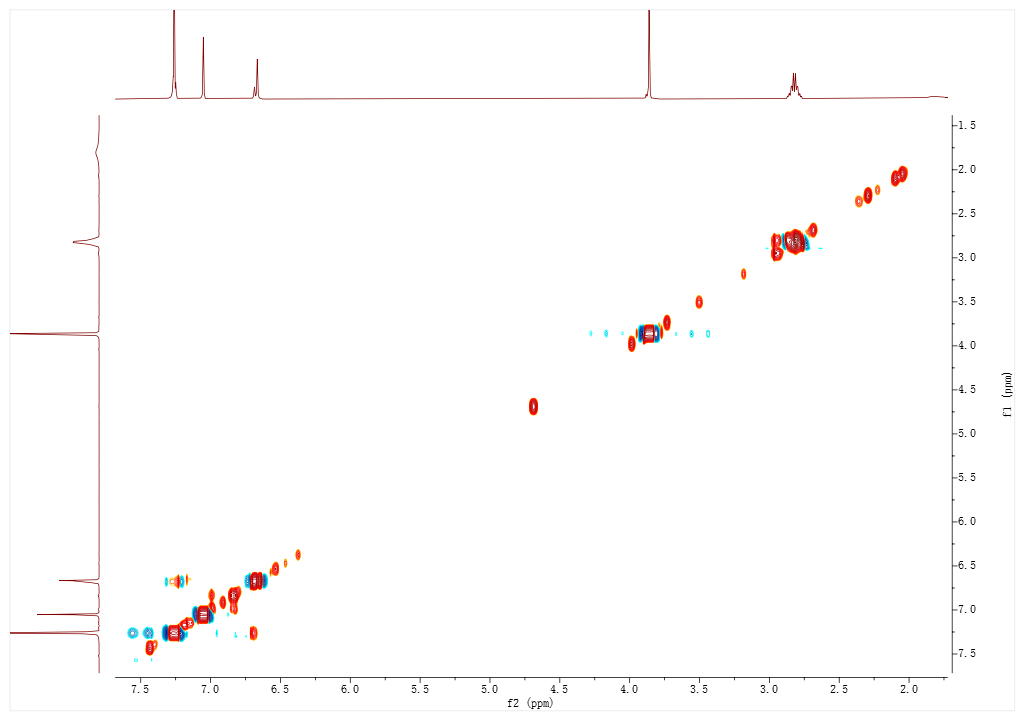


Figure S41. HSQC Spectrum of Compound **5** in CDCl_3_

Figure S42. HMBC Spectrum of Compound **5** in CDCl_3_

Figure S43. (-) Total HRESIMS Spectrum of Compound **5**


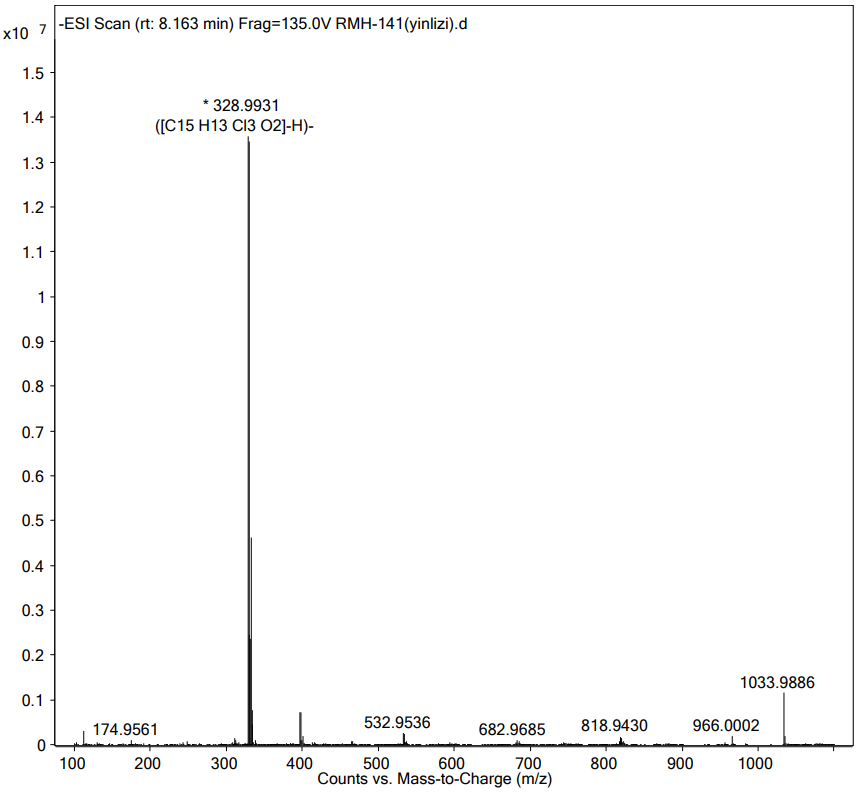


Figure S44. (-) Partial HRESIMS Spectrum of Compound **5**


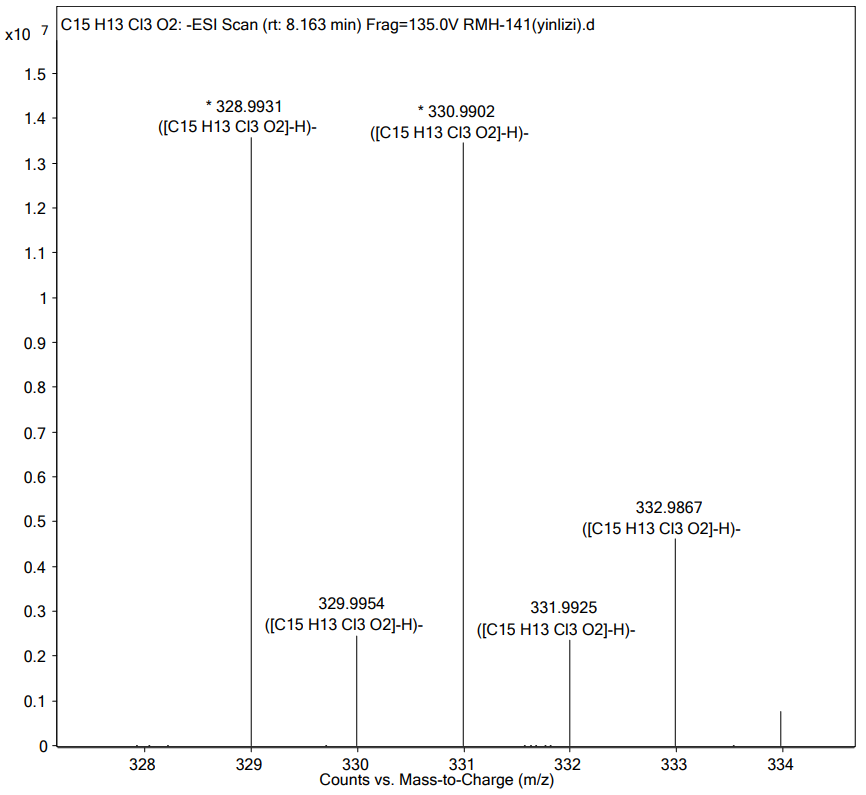


Figure S45. UV Spectrum of Compound **5**


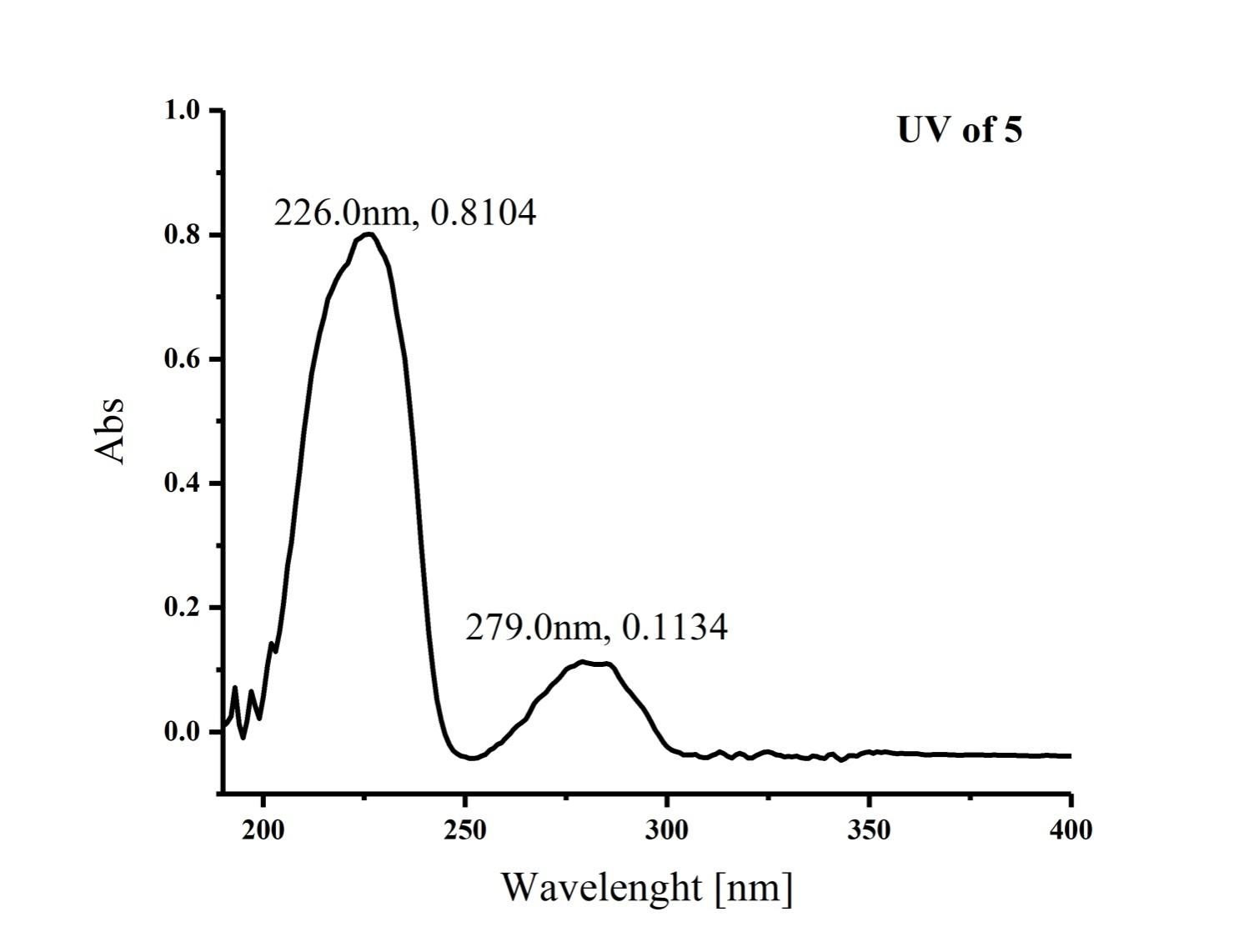


Figure S46. IR (KBr disc) Spectrum of Compound **5**


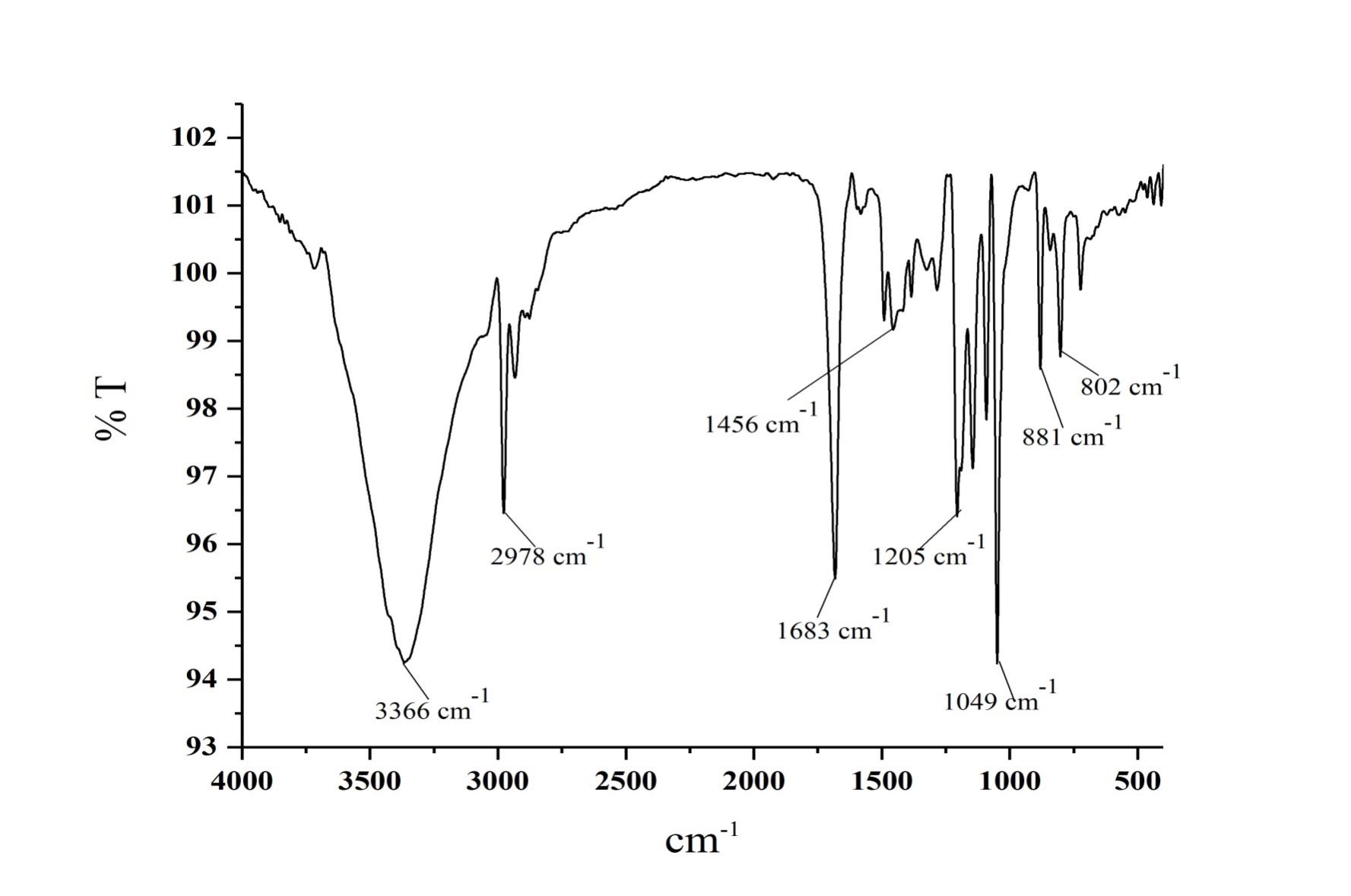


Figure S47. ^1^H NMR Spectrum of Compound **6** in CDCl_3_


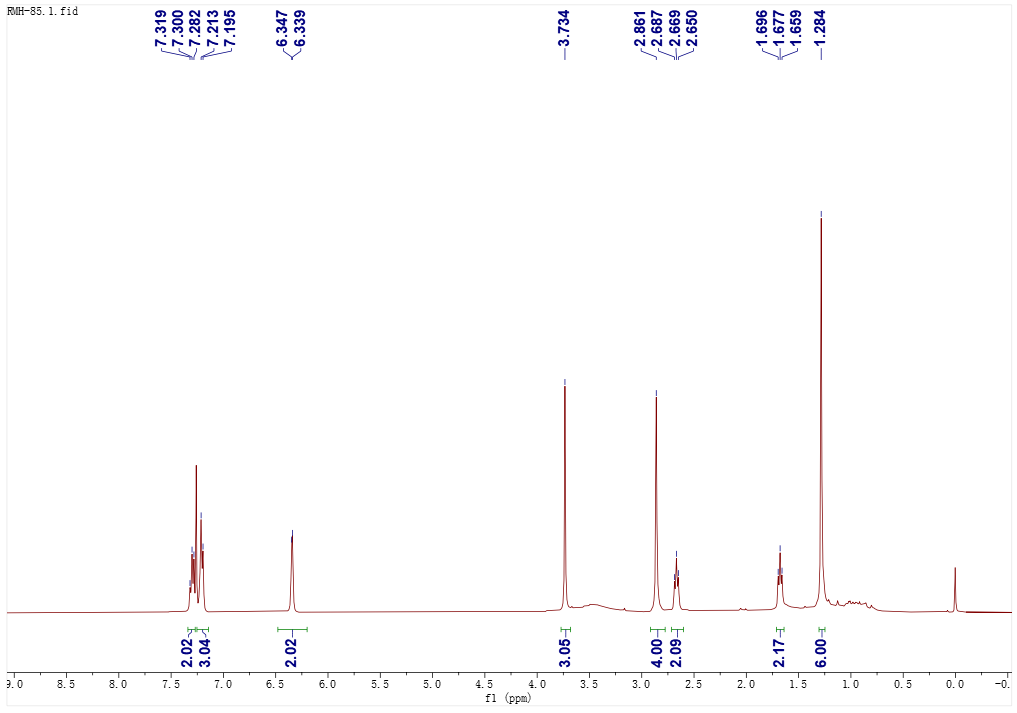


Figure S48. ^13^C NMR Spectrum of Compound **6** in CDCl_3_


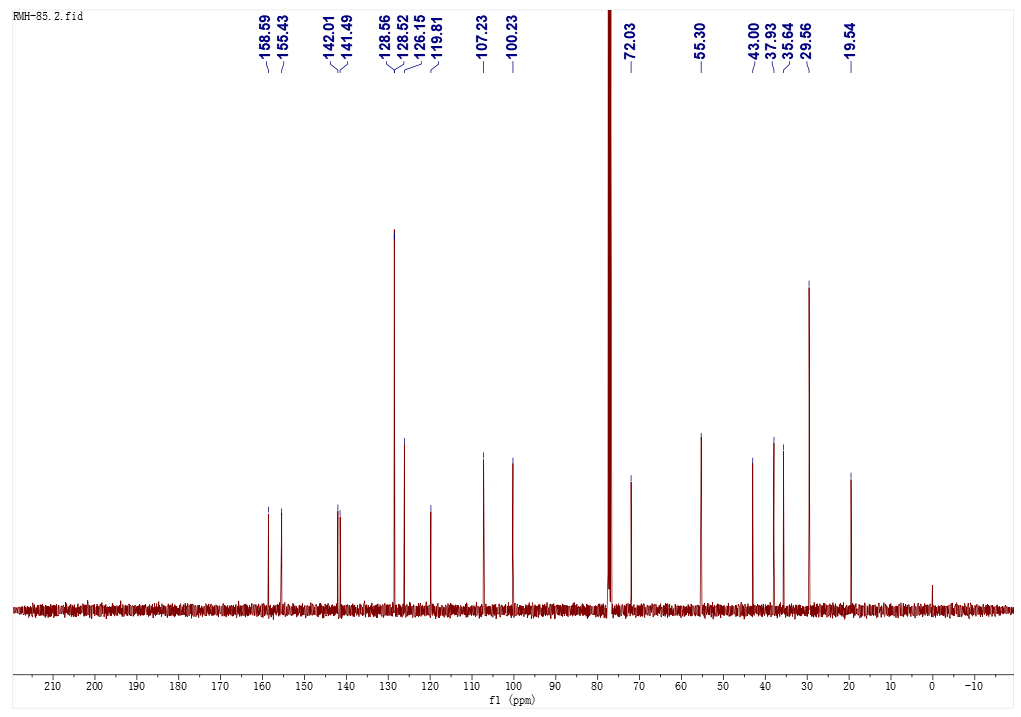


Figure S49. (+) HRESIMS Spectrum of Compound **6**


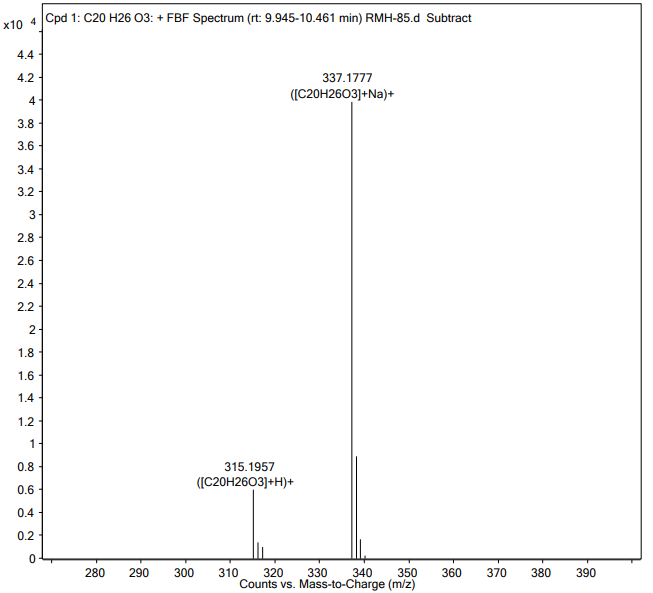


Figure S50. ^1^H NMR Spectrum of Compound 7 in CDCl_3_


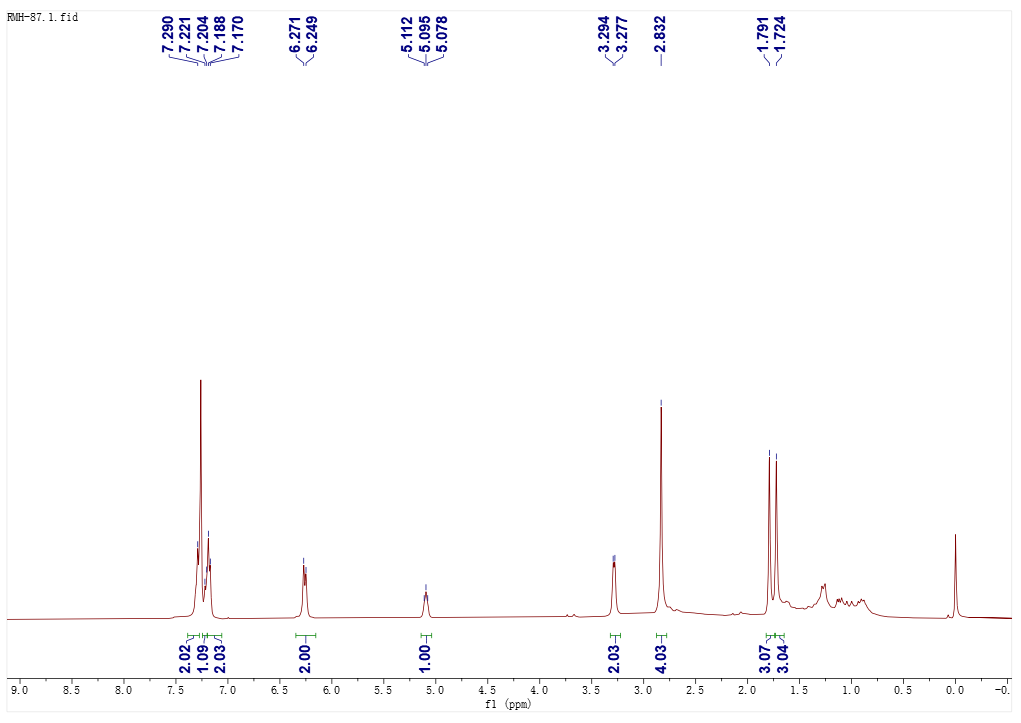


Figure S51. ^13^C NMR Spectrum of Compound **7** in CDCl_3_


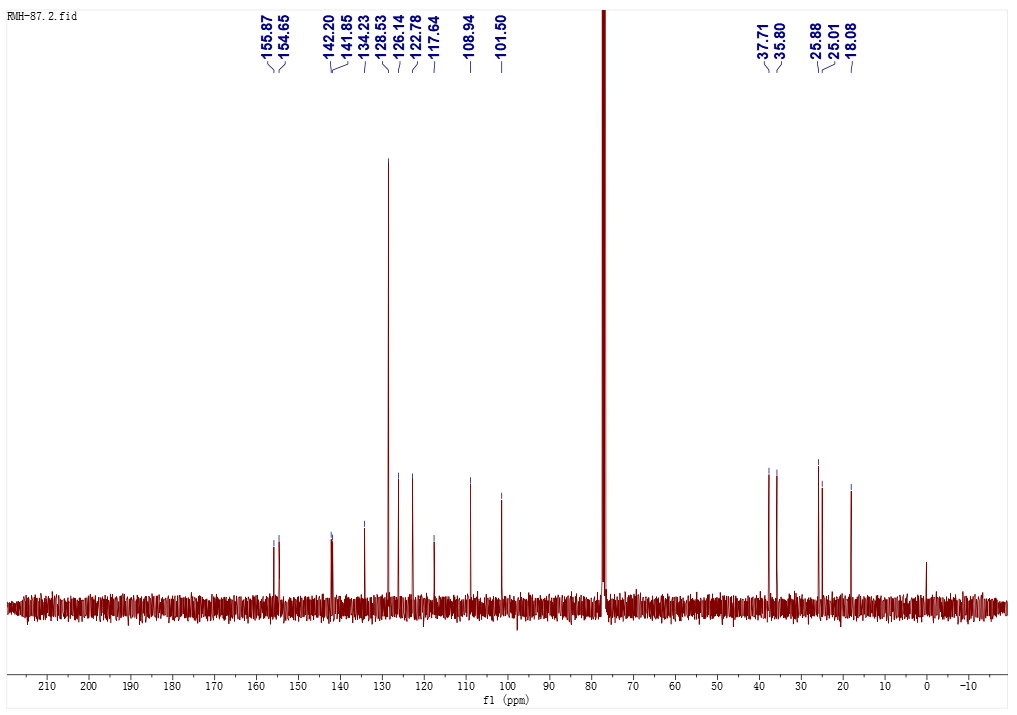


Figure S52. (+) HRESIMS Spectrum of Compound **7**


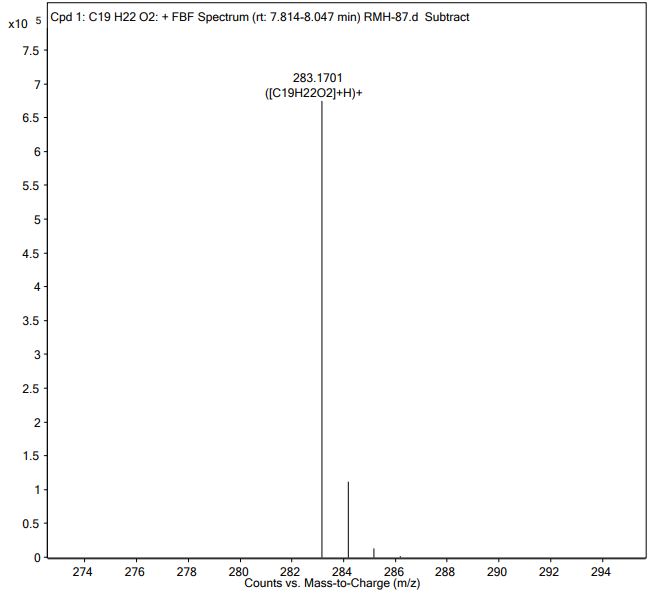


Figure S53. ^1^H NMR Spectrum of Compound **8** in MeOH-*d*_4_


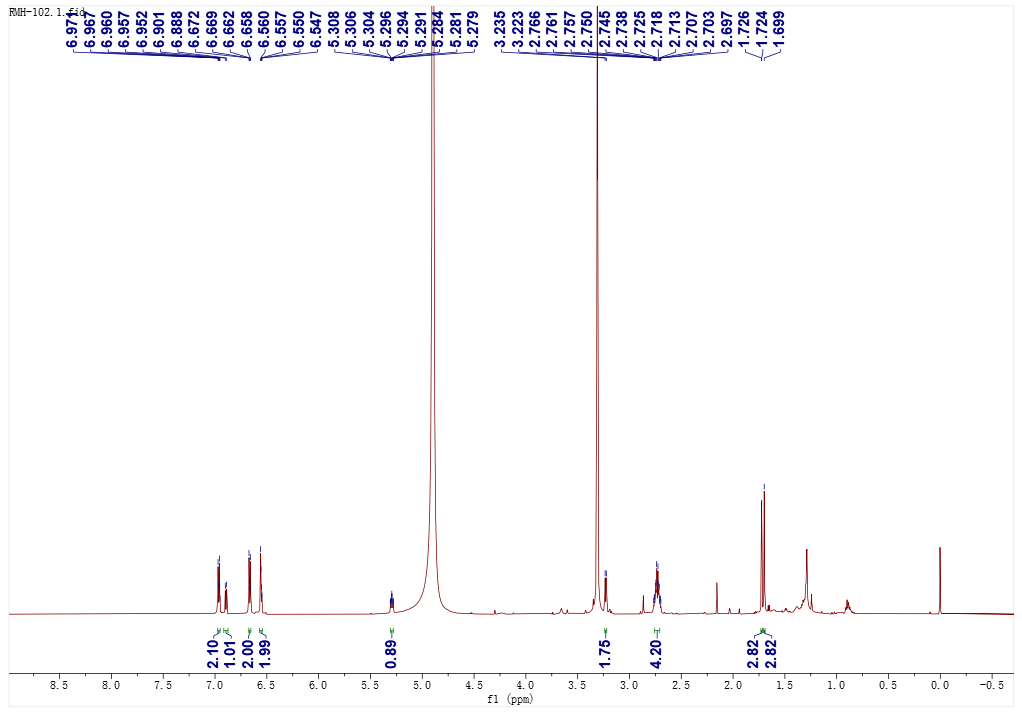


Figure S54. ^13^C NMR Spectrum of Compound **8** in MeOH-*d*_4_


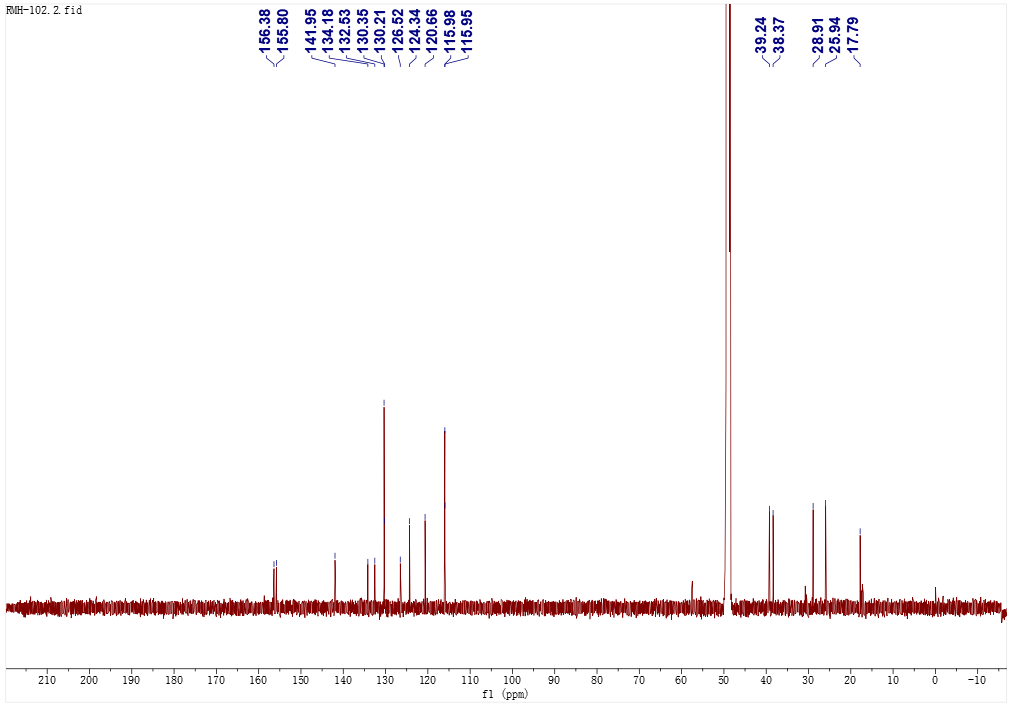


Figure S55. ^1^H-^1^H COSY Spectrum of Compound **8** in MeOH-*d*_4_


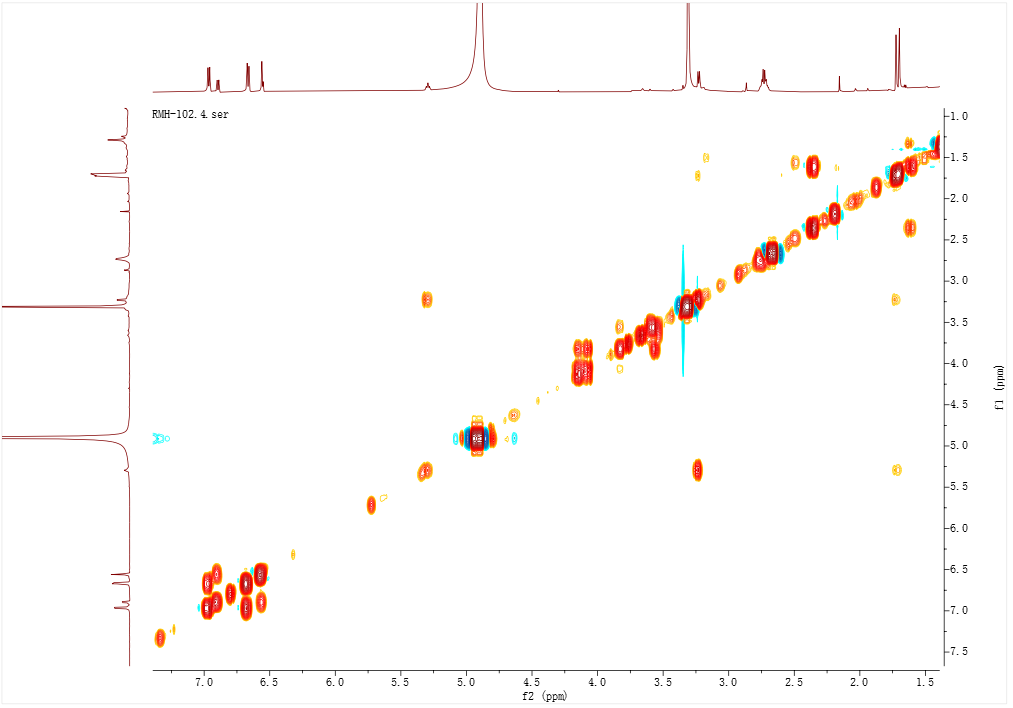


Figure S56. HSQC Spectrum of Compound **8** in MeOH-*d*_4_


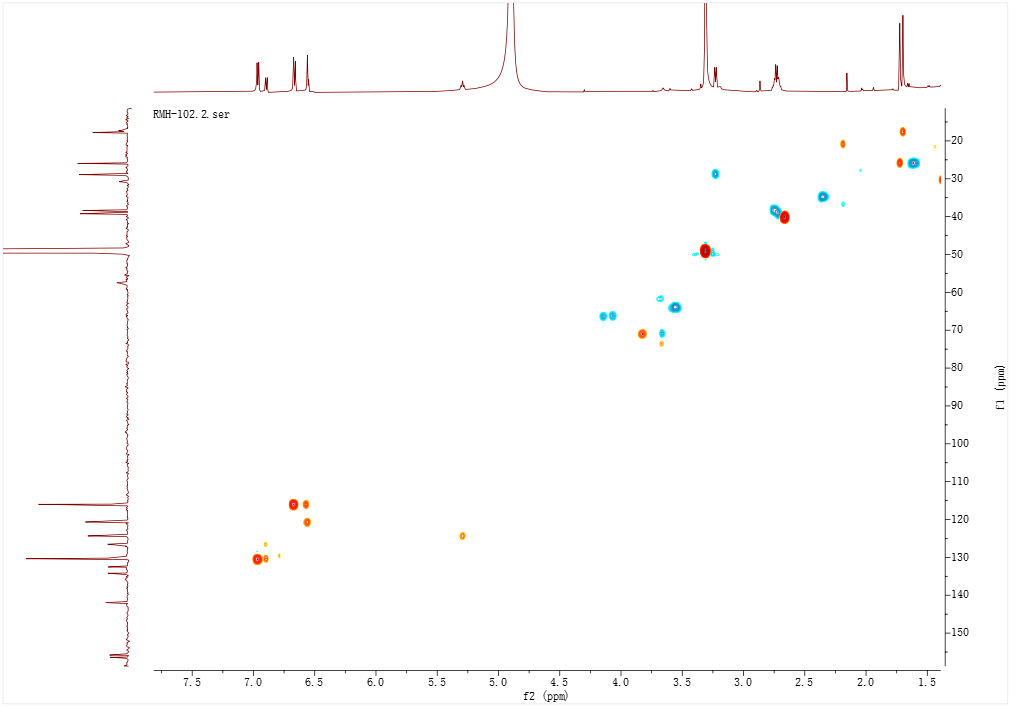


Figure S57. HMBC Spectrum of Compound **8** in MeOH-*d*_4_


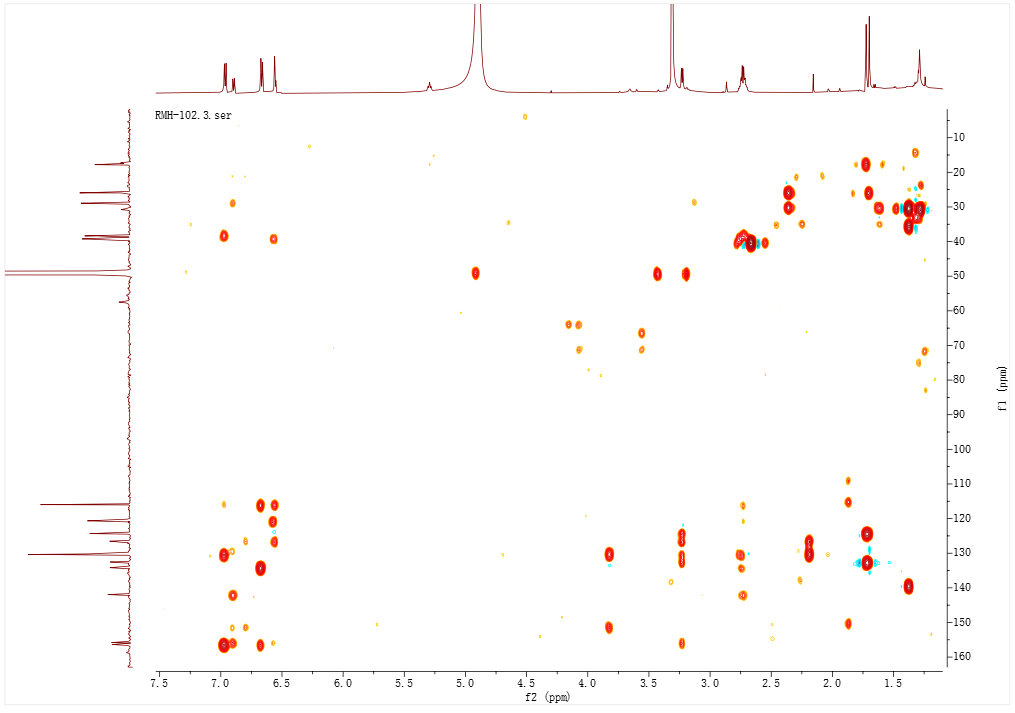


Figure S58. (+) HRESIMS Spectrum of Compound **8**


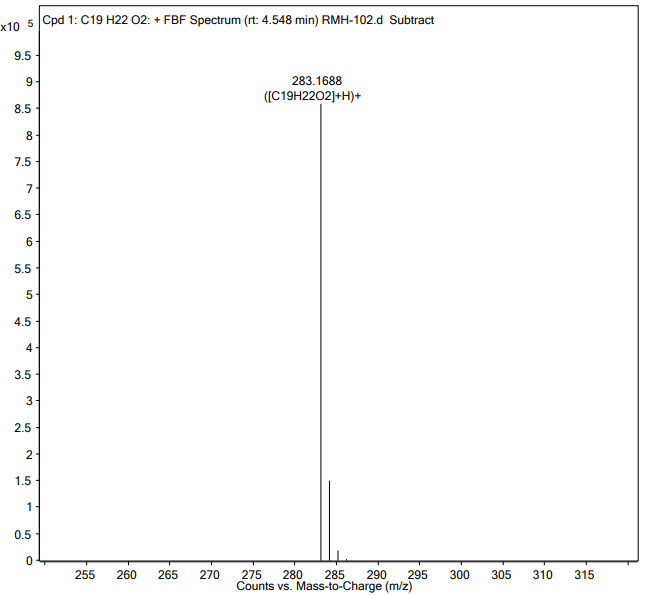


Table S3.**^1^H and ^13^C NMR Data of Compound 8**

| No | *δ*_H_, mult. (*J*) | *δ*_C_, type |
| --- | --- | --- |
| 1 |  | 142.0, s |
| 2 | 6.56, m^b^ | 116.0, d |
| 3 |  | 155.8, s |
| 4 |  | 126.5, s |
| 5 | 6.89, d (7.8) | 130.2, d |
| 6 | 6.55, m^b^ | 120.7, d |
| 1′ | 3.23, d (7.2) | 28.9, t |
| 2′ | 5.29, m | 124.3, d |
| 3′ |  | 132.6, s |
| 4′ | 1.72, s | 25.9, q |
| 5′ | 1.70, s | 17.8, q |
| α | 2.74, m^b^ | 39.2, t |
| β | 2.74, m^b^ | 38.4, t |
| 1′′ |  | 134.2, s |
| 2′′ | 6.96, m | 130.4, d |
| 3′′ | 6.66, m | 116.0, d |
| 4′′ |  | 156.4, s |
| 5′′ | 6.66, m | 116.0, d |
| 6′′ | 6.96, m | 130.4, d |

^b^Overlapped signals are reported without designating multiplicity.
